# Supplementary material for: Multiple, independent colonizations of the Hawaiian Archipelago by the family Dolichopodidae (Diptera)
Source: PeerJ. 2016 Nov 17;4:e2704. doi: 10.7717/peerj.2704 (PMC5119231; doi:10.7717/peerj.2704)

Appendix S1. Taxonomic sampling, GenBank accession numbers, partitioning and supplementary trees.

TABLE S1A. TAXON SAMPLING AND GENBANK ACCESSION NUMBERS FOR *EURYNOGASTER* COMPLEX, *CONCHOPUS,* AND NON-HAWAIIAN ENDEMIC DOLICHOPODIDAE INCLUDED IN THIS STUDY.

Genus	*species*	Locality	LabelName	ND2	COI	COII	12s	16s	CAD	EF1aA	EF1aB
**Eurynogaster complex specimens**											
Adachia	*apicenigra*	HAWAII, Olaa Forest, Pole 44	Ad.apicenigra107	KX781491	KX781446	KX781540	KX781592	KX781644	KX781396		KX808417
Adachia	*hispida*	HAWAII, Olaa Forest, Puu Unit, 4300'	Ad.hispida200978	KX781492	KX781447	KX781541	KX781593	KX781645	KX781397	KX808394	
Adachia	*n.sp.nr.hispida*	KAUAI, Kawaikoi Stream, 3500', 18 May 2007, sweeping over stream	Ad.sp.nr.hispida205015	KX781493	KX781448	KX781542	KX781594	KX781646	KX781398	KX808395	KX808418
Arciellia	*dolichostoma*	KAUAI, Puu O Kila Road, 4080'	Ar.dolichostoma205016	KX781494	KX781449	KX781568	KX781595	KX781647	KX781399	KX808396	KX808419
Arciellia	*dolichostoma*	KAUAI, Pihea Trail, 3600'	Ar.dolichostoma205017	KX781495	KX781450	KX781569	KX781596	KX781648	KX781400	KX808397	KX808420
Arciellia	*dolichostoma*	MAUI, Haleakala NP, Kaupo Trail	Ar.dolichostoma205359	KX781496	KX781451	KX781572	KX781597	KX781649			KX808421
Arciellia	*xanthopleura*	KAUAI, Kokee SP, Nualolo Tr.	Ar.xanthopleura147			KX781570	KX781598	KX781650	KX781401		KX808422
Arciellia	*xanthopleura*	KAUAI, Pihea Trail, 3600'	Ar.xanthopleura205018	KX781497	KX781452	KX781571	KX781599	KX781651	KX781402	KX808398	KX808423
Eurynogaster	*cilifemorata*	OAHU, Poamoho, 1.5 mi.	E.cilifemorata94	KX781512		KX781561	KX781613	KX781664	KX781416		KX808436
Eurynogaster	*clavaticauda*	OAHU, Poamoho, stream	E.clavaticauda95	KX781513	KX781466	KX781554	KX781614	KX781665	KX781417	KX808403	KX808437
Eurynogaster	*kauaiensis*	KAUAI, Pihea Trail	E.kauaiensis131	KX781514		KX781566	KX781615	KX781666	KX781418		KX808438
Eurynogaster	*maculata*	OAHU, Poamoho Tr.	E.maculata132	KX781515	KX781467	KX781556	KX781616	KX781667	KX781419		KX808439
Eurynogaster	*maculata*	MAUI, Paliku, crater wall, 6600'	E.maculata205058_205380	KX781516	KX781468	KX781557	KX781617	KX781668	KX781420	KX808404	KX808440
Eurynogaster	*n.sp.A*	HAWAII, Hakalau	E.n.sp.A133	KX781517		KX781549	KX781618	KX781669	KX781421		KX808441
Eurynogaster	*n.sp.B*	OAHU, Mt. Kaala	E.n.sp.B105			KX781562	KX781621	KX781672	KX781424		KX808444
Eurynogaster	*n.sp.C*	OAHU, Poamoho Tr.	E.n.sp.C128	KX781518	KX781469	KX781563	KX781619	KX781670	KX781422	KX808405	KX808442
Eurynogaster	*paludis*	MAUI, Pihea Trail, 3600'	E.paludis205025	KX781519	KX781470	KX781567	KX781620	KX781671	KX781423	KX808406	KX808443
Eurynogaster	*sp.nr.cilifemorata*	OAHU, Mt. Kaala	E.sp.nr.cilifemorata102			KX781560		KX781673			

Eurynogaster	*sp.nr.cilifemorata*	MAUI, Haiku Uka, Heed Trail, 4200'	E.sp.nr.cilifemorata205049	KX781520	KX781471	KX781559	KX781622	KX781674	KX781425	KX808407	KX808445
Eurynogaster	*nr. cilifemorata*	MAUI, East Maui Irrigation, Haiku Uka,Heed Trail	E.sp.nr.cilifemorata83	KX781521	KX781472	KX781552	KX781623	KX781675	KX781426	KX808408	KX808446
Eurynogaster	*sp.nr.clavaticauda*	OAHU, Poamoho, stream	E.sp.nr.clavaticauda93	KX781522	KX781473	KX781555	KX781624	KX781676	KX781427	KX808409	
Eurynogaster	*sp.nr.hawaiiensis*	MAUI, Haiku Uka,Carson Trail, 4200'	E.sp.nr.hawaiiensis205031	KX781523	KX781474	KX781564	KX781625	KX781677	KX781428	KX808410	KX808447
Eurynogaster	*sp.nr.hawaiiensis*	MAUI, East Maui Irrigation, Haiku Uka, Carson Trail	E.sp.nr.hawaiiensis80	KX781524	KX781475	KX781565	KX781626	KX781678	KX781429	KX808411	KX808448
Eurynogaster	*sp.nr.maculata*	HAWAII, Hakalau	E.sp.nr.maculata115			KX781547	KX781627	KX781679			
Eurynogaster	*sp.nr.maculata*	HAWAII, Kohalas, Puu O Uni NAR	E.sp.nr.maculata126	KX781525	KX781476	KX781550	KX781628	KX781680	KX781430		KX808449
Eurynogaster	*sp.nr.maculata*	MAUI, Trail to Kawiki, 1 mi. from Paliku cabin	E.sp.nr.maculata141	KX781526	KX781477	KX781558	KX781629	KX781681	KX781431		KX808450
Eurynogaster	*sp.*	HAWAII, Olaa Forest, Pole 44	E.sp112	KX781527	KX781478	KX781548	KX781630	KX781682	KX781432	KX808412	
Eurynogaster	*sp.*	MAUI, Waikamoi Forest Preserve, Heed Trail	E.sp135	KX781528	KX781479	KX781553	KX781631	KX781683	KX781433		KX808451
Eurynogaster	*variabilis*	HAWAII, Kau FR, Alahi Spring	E.variabilis205226	KX781529	KX781480	KX781551		KX781684	KX781434		KX808452
Elmoia	*bullata*	HAWAII, Kohala State Forest, trail to Waimanu Stream	El.bullata205260	KX781530		KX781543	KX781632	KX781685	KX781435	KX808413	KX808453
Elmoia	*saxatilis*	OAHU, Mt. Kaala	El.saxatilis106		KX781481	KX781546	KX781633	KX781686	KX781436		KX808454
Major	*minor*	OAHU, Mt. Kaala	M.minor205097_111	KX781531	KX781482	KX781544	KX781634	KX781687	KX781437		KX808455
Sigmatineurum	*n.sp.D*	HAWAII, Kohala Mountains	Si.n.sp.D205265	KX781532	KX781483	KX781545	KX781635	KX781688	KX781438		KX808456
Sweziella	*tergoprolixa*	MAUI, Haiku Uka, Heed Trail, 4200'	Sw.tergoprolixa205048	KX781533	KX781484	KX781578	KX781636	KX781689	KX781439	KX808414	KX808457
Uropachys	*clavastyla*	KAUAI, Kokee SP, Nualolo Tr.	U.clavastyla130		KX781486	KX781575	KX781639	KX781692	KX781441		KX808459
Uropachys	*fusticercus*	KAUAI, Na Pali-Kona FR Pihea Trail	U.fusticercus205027	KX781536	KX781487	KX781573	KX781640	KX781693	KX781442	KX808416	KX808460
Uropachys	*fusticercus*	KAUAI, Na Pali-Kona FR Pihea Trail	U.fusticercus61	KX781537	KX781488	KX781574	KX781641	KX781694	KX781443		KX808461
Uropachys	*palustricola*	KAUAI, Alakai Swamp GB27A	U.palustricola127	KX781538	KX781489	KX781576	KX781642	KX781695	KX781444		KX808462

Uropachys	*palustricola*	KAUAI, Pihea Trail	U.palustricola134	KX781539	KX781490	KX781577	KX781643	KX781696	KX781445		KX808463
**Other Hawaiian endemic Dolichopodidae specimens**											
Conchopus	*ciliatus*	HAWAII, Whittington Beach Park, South Coast, Kau	Conchopus.ciliatus96	KX781504	KX781459	KX781590		KX781656	KX781408		KX808428
Conchopus	*pacificus*	HAWAII, Whittington Beach Park, South Coast, Kau	Conchopus.pacificus122	KX781505	KX781460	KX781591	KX781606	KX781657	KX781409		KX808429
Conchopus	*acrostichalis*	OAHU, Haleiwa Beach	Conchopus.acrostichalis92	KX781503	KX781458	KX781589	KX781605	KX781655	KX781407		KX808427
Thinophilus	*hardyi*	HAWAII, Whittington Beach Park, South Coast, Kau	Th.hardyi205118	KX781535		KX781588	KX781638	KX781691			
**Non-Hawaiian endemic Dolichopodidae specimens**											
Chrysotus	*longipalpis*	OAHU, Manoa Cliff Trail, 1800'	Ch.longipalpis205060	KX781498	KX781453	KX781579	KX781600	KX781652	KX781403		KX808424
Chrysotus	*longipalpis*	OAHU, Honolulu Watershed FR, Manoa Cliffs Trail	Ch.longipalpis205379	KX781499	KX781454	KX781581	KX781601	KX781653	KX781404		KX808425
Chrysotus	*sp*	MARQUESAS, HIVA OA	Ch.sp173	KX781502	KX781457		KX781604				
Chrysotus	*sp nr.longipalpis*	MAUI, Kahanaiki Gulch, 2100'	Ch.sp.nr.longipalpis205030	KX781500	KX781455		KX781602		KX781405		KX808426
Chrysotus	*sp.nr.longipalpis*	MAUI, Puu Kukui, Kahanaiki Gulch	Ch.sp.nr.longipalpis85	KX781501	KX781456	KX781580	KX781603	KX781654	KX781406		
Condylostylus	*sp.*	OAHU, Maunaloa Beach Rocks	Condylostylus.sp164	KX781506	KX781461		KX781607	KX781658	KX781410		KX808430
Dolichopus	*exsul*	OAHU, Poamoho	D.exsul169	KX781507		KX781585	KX781608	KX781659	KX781411		KX808431
Dolichopus	*exsul*	KAUAI, Pihea Trail, 3500'	D.exsul205024	KX781509	KX781462	KX781583	KX781609	KX781660	KX781412	KX808399	KX808432
Dolichopus	*exsul*	MAUI, Puu Kukui Trail, 2900-3700'	D.exsul205034	KX781508	KX781463	KX781582	KX781610	KX781661	KX781413	KX808400	KX808433
Dolichopus	*exsul*	HAWAII, Kau FR, Alahi Spring	D.exsul205232	KX781510	KX781464	KX781584	KX781611	KX781662	KX781414	KX808401	KX808434
Dolichopus	*exsul*	HAWAII, Kohala Mountains	D.exsul205282	KX781511	KX781465	KX781586	KX781612	KX781663	KX781415	KX808402	KX808435
Tachytrechus	*angustipennis*	KAUAI, Kumuwela Trail, 3500'	T.angustipennis205026	KX781534	KX781485	KX781587	KX781637	KX781690	KX781440	KX808415	KX808458

TABLE S1B. SUMMARY OF DATA PARTITIONS AND NUCLEOTIDE MODELS DETERMINED FOR EACH PARTITION, SELECTED USING BIC IN PARTITIONFINDER (LANFEAR ET AL., 2012).

**Program (# partitions)**	**Figure**	**Data partitions: nucleotide models selected by PartitionFinder**
Dataset A<br>Dolichopodidae<br><br>Mr Bayes/<br>RAxML(11)	Figures 2, S1a & S1b in Appendix 1	**Mr Bayes:**<br>COI_3, COII_3, ND2_3: GTR+I+G<br>COI_1, COII_1: GTR+I+G<br>EF1_2, COI_2, COII_2: GTR+I+G<br>ND2_1: GTR+G<br>ND2_2: GTR+G<br>12S, 16S: GTR+I+G<br>CAD_3: HKY+I+G<br>CAD_1, CAD_2: K80+I+G<br>CADintron: GTR+I+G<br>EF1_3: HKY+G<br>EF1_1: F81+I+G<br><br>**RAxML:**<br>Each partition was run using the GTR+GAMMA model
Dataset B<br>(*Eurynogaster* complex):<br><br>Mr Bayes/<br>RAxML(11)	Figure 1 & Figure S1c-l in Appendix 1	**Mr Bayes:**<br>CAD_3: HKY+I<br>CAD_1: GTR+G<br>CAD_2, EF1A_2, ND_2: HKY+I+G<br>CADintron, EF1Aintron1, EFAintron2: HKY+I<br>EF1A_3, EF1B_3: HKY+G<br>EF1A_1, EF1B_1: F81+I<br>COI_2, COII_2, EF1B_2: F81+I+G<br>EF1Bintron: F81<br>12S, 16S, ND2_1: GTR+I+G<br>COI_3, COII_3, ND2_3: GTR+I+G<br>COI_1, COII_1: GTR+G<br><br>**RAxML:**<br>Each partition was run using the GTR+GAMMA model
Dataset B<br>(*Eurynogaster* complex):<br><br>BEAST:<br>Island calibrations<br>(10)	Figures 1 & S2b in Appendix 2	CAD_3: HKY<br>CAD_1, EF1A_1, EF1B_1: TrN+I<br>CAD_2, EF1A_2, ND_2: HKY+I+G<br>CADintron, EF1Aintron1, EF1Aintron2: HKY+I<br>EF1A_3, EF1B_3: HKY+G<br>COI_2, COII_2, EF1B_2: HKY+I+G<br>EF1Bintron: HKY<br>12S, 16S, ND2_1: GTR+I+G<br>COI_3, COII_3, ND2_3: GTR+I+G<br>COI_1, COII_1: GTR+G
Dataset B<br>(*Eurynogaster* complex):<br><br>BEAST:<br>Divergence rates (11)	Figures S2c & S2d in Appendix 2	COI: GTR+I+G<br>CAD_3: HKY+I<br>CAD_1, EF1A_2, EF1B_1: TrN+I<br>CAD_2, EF1A_2, ND2_2: HKY+I+G<br>CADintron, EF1Aintron1, EF1Aintron2: HKY+I<br>EF1A_3, EF1B_3: HKY+G<br>COII_2, EF1B_2: HKY+I<br>EF1Bintron: HKY<br>12S, 16S, ND2_1: GTR+I+G<br>COII_3, ND2_3: GTR+I+G<br>COII_1: TrN+G

SUPPLEMENTARY TREES:

FIGURE S1A. COMBINED DOLICHOPODIDAE ANALYSIS (DATA SET A). Majority rule consensus tree summarizing Bayesian analysis, with the *Eurynogaster* complex and *Campsicnemus* collapsed. Bayesian posterior probabilities (Mr. Bayes) and bootstrap supports from the maximum likelihood analysis (RAxML) are displayed as boxes.

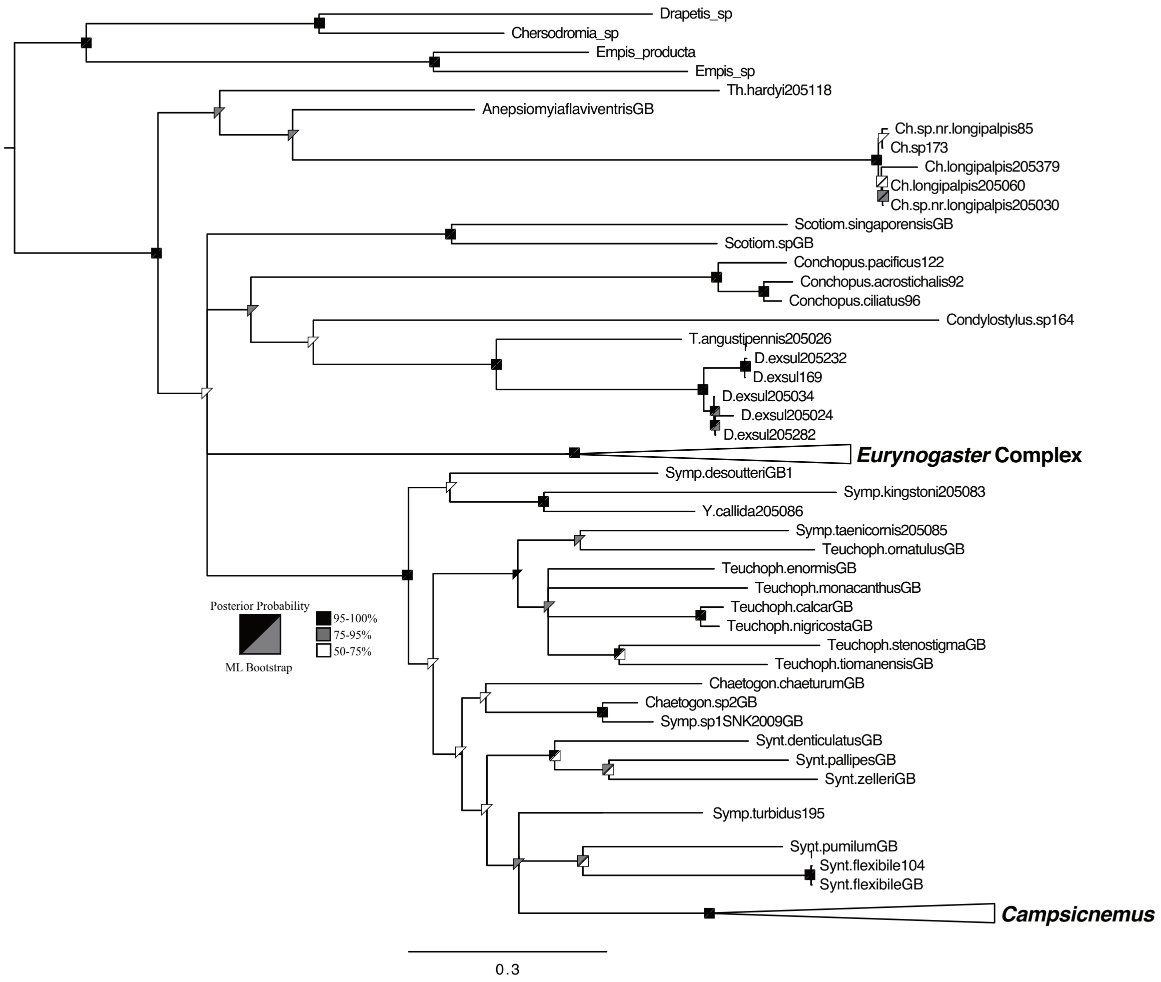

FIGURE S1B. MAXIMUM LIKELIHOOD ANALYSIS OF THE DOLICHOPODIDAE (DATA SET A).

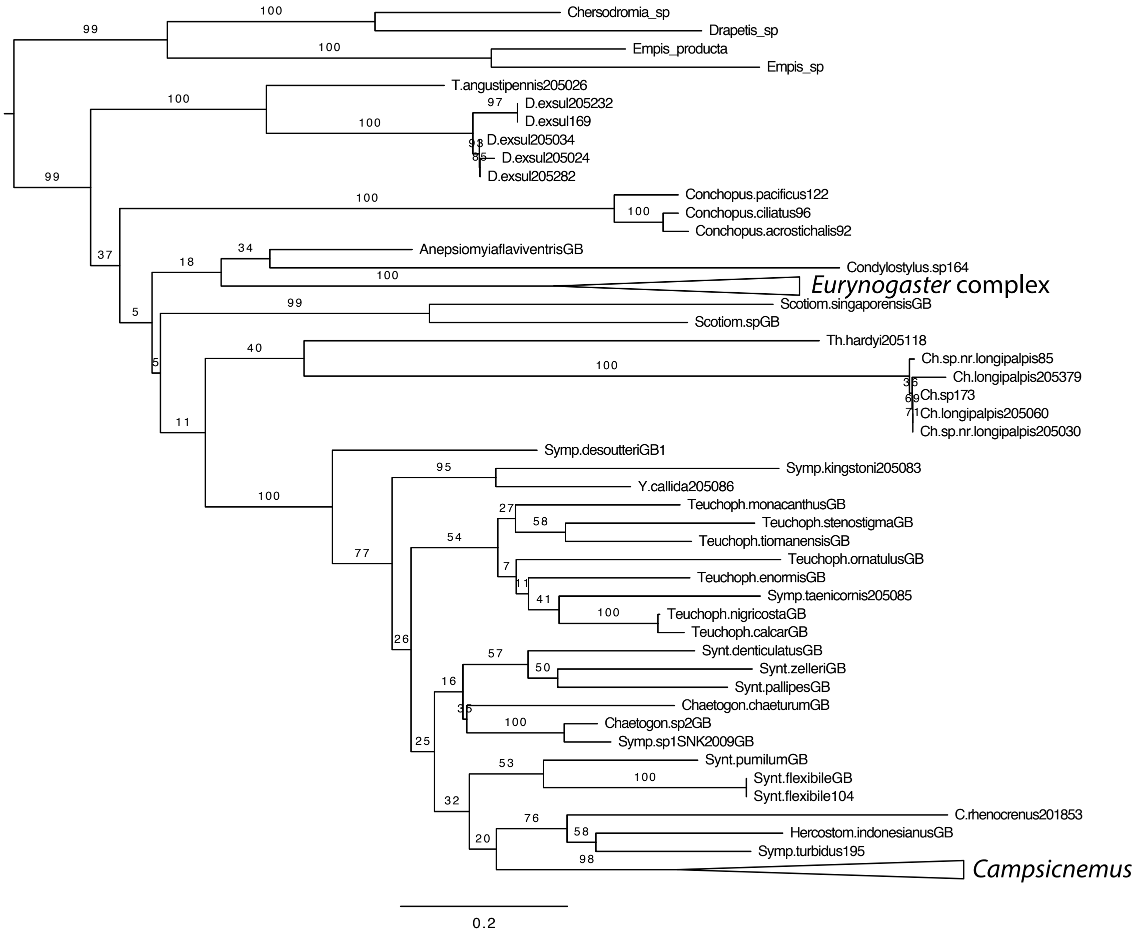

FIGURE S1C. Majority rule consensus tree summarizing Bayesian analysis of the *Eurynogaster* complex. Bayesian posterior probabilities (Mr. Bayes) and bootstrap supports from the maximum likelihood analysis (RAxML) are displayed as boxes. Islands that each specimen was collected from are shown next to each tip.

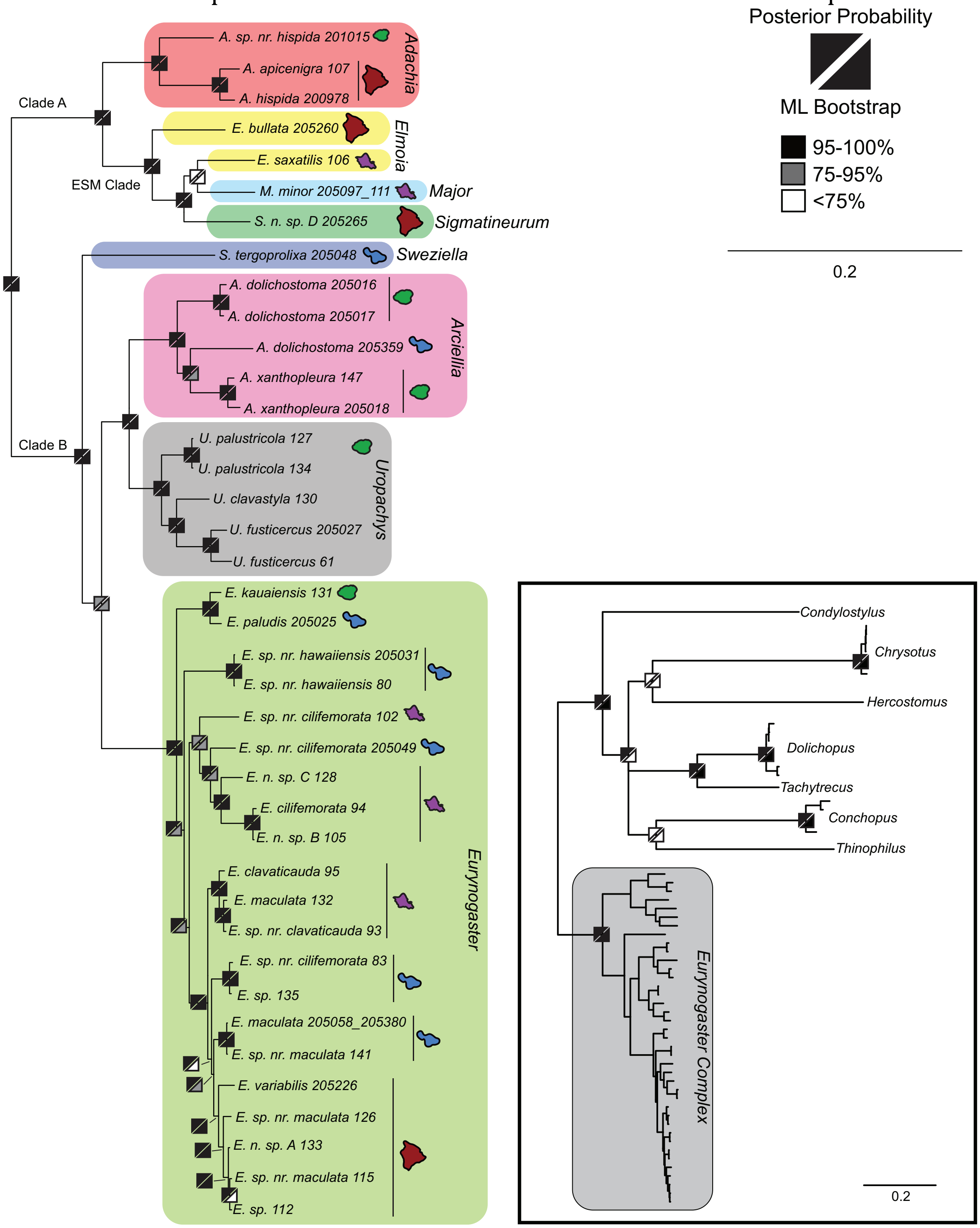

FIGURE S1D. COMBINED MAXIMUM LIKELIHOOD ANALYSIS OF ALL GENES
(DATA SET B).

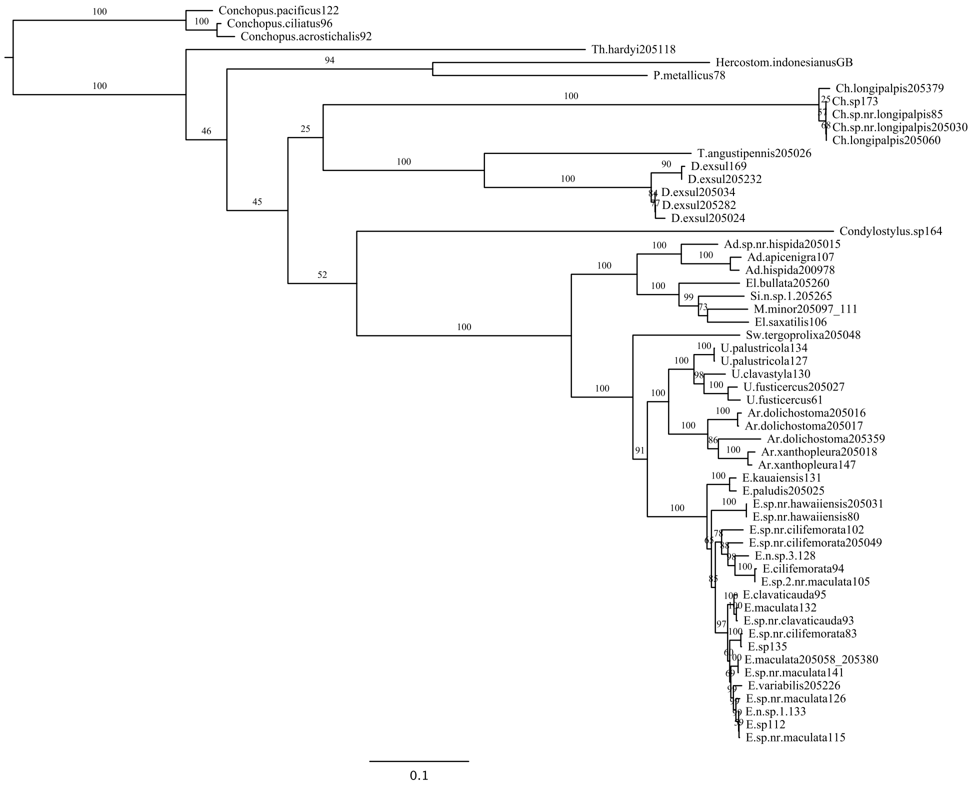

SUPPLEMENTARY TREES: INDIVIDUAL MAXIMUM LIKELIHOOD GENE TREES:

FIGURE S1E. COI

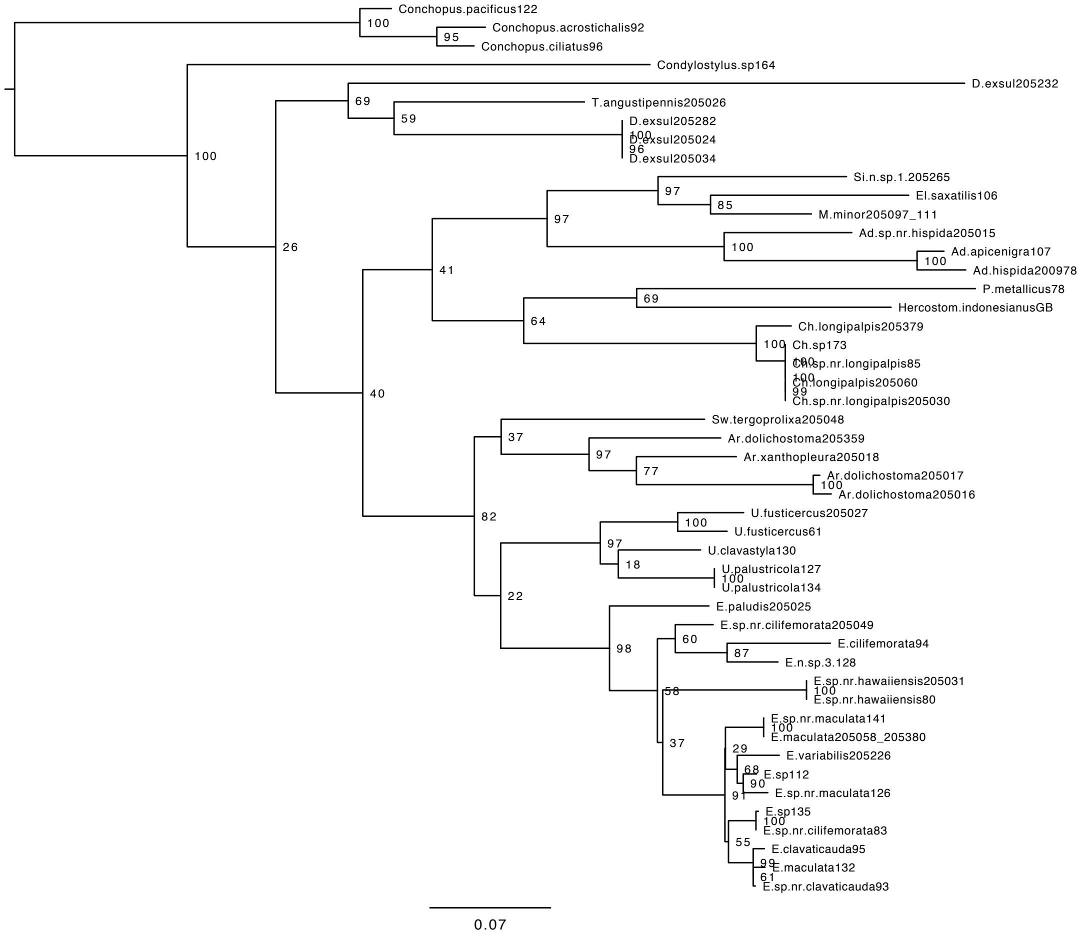

FIGURE S1F. COII

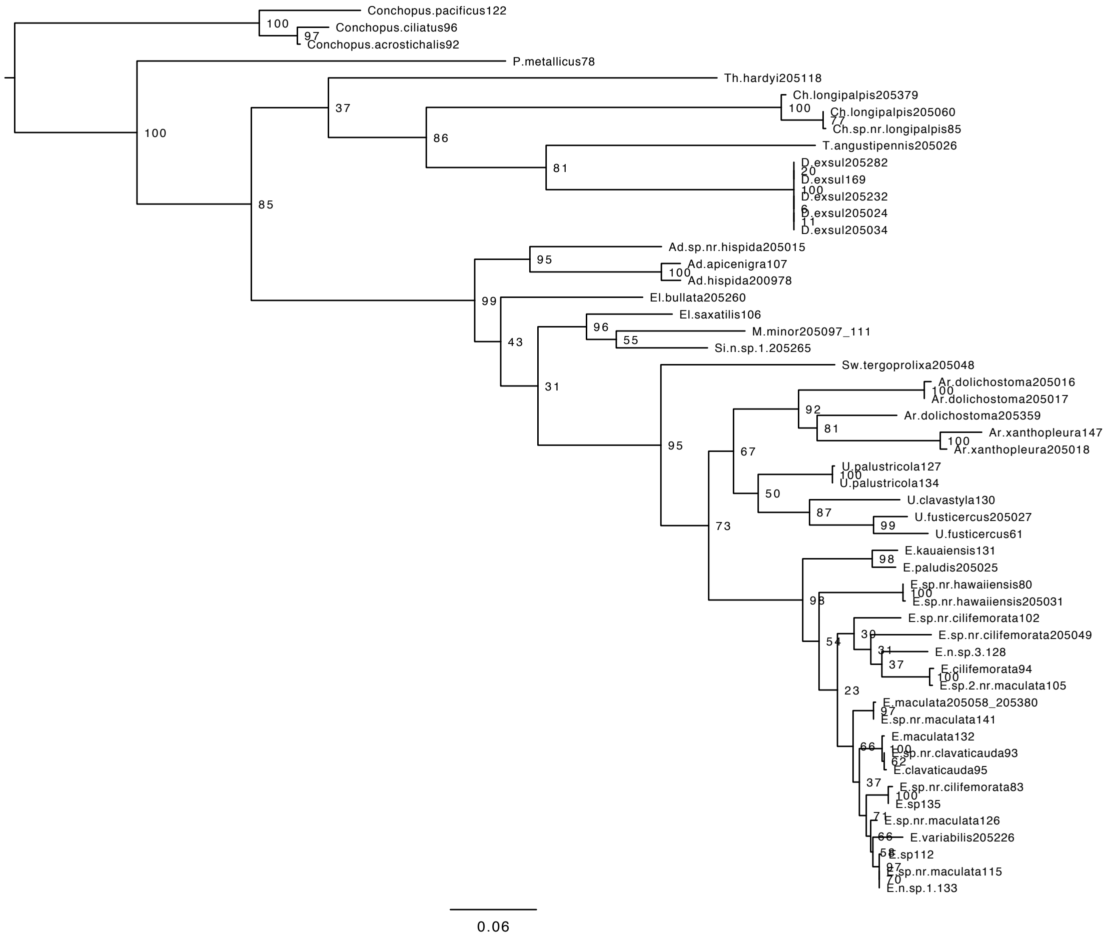

FIGURE S1G. ND2

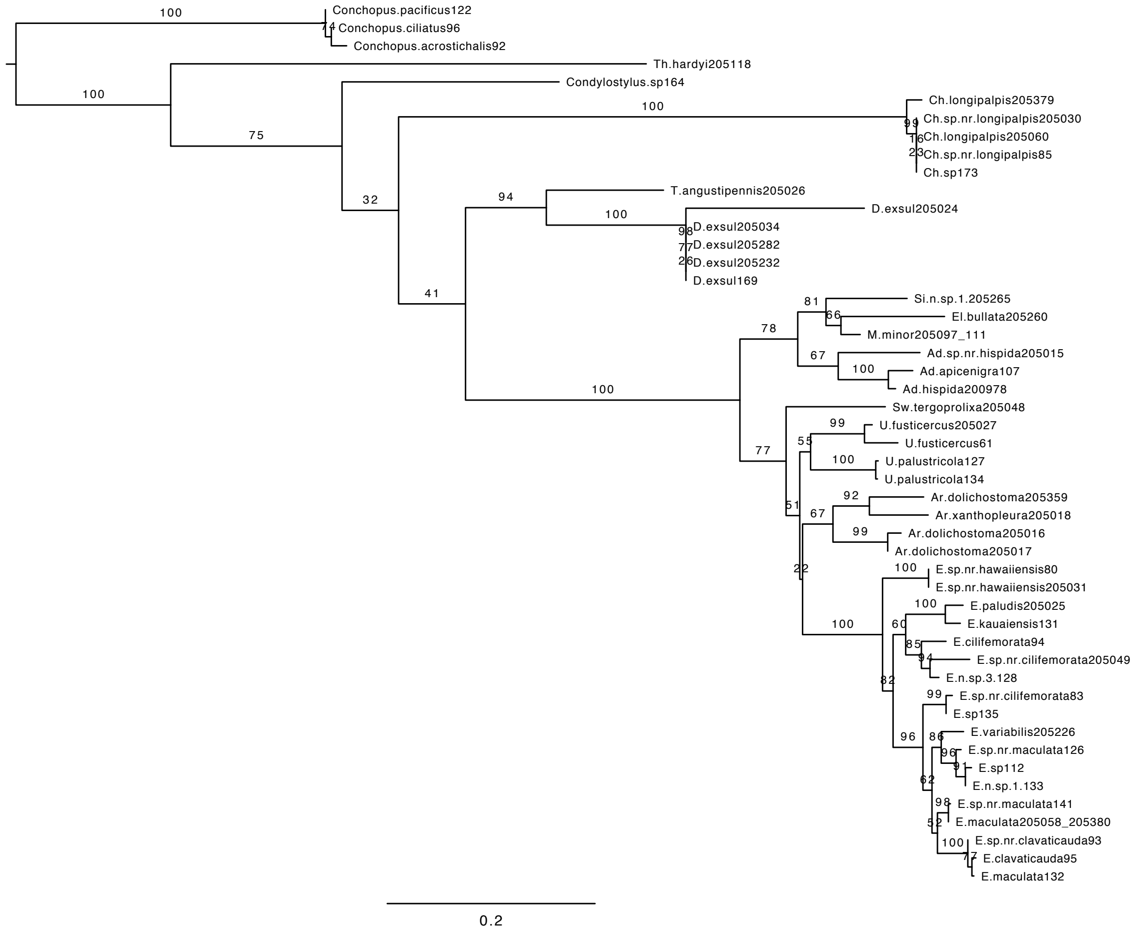

Figure S1h. 12S

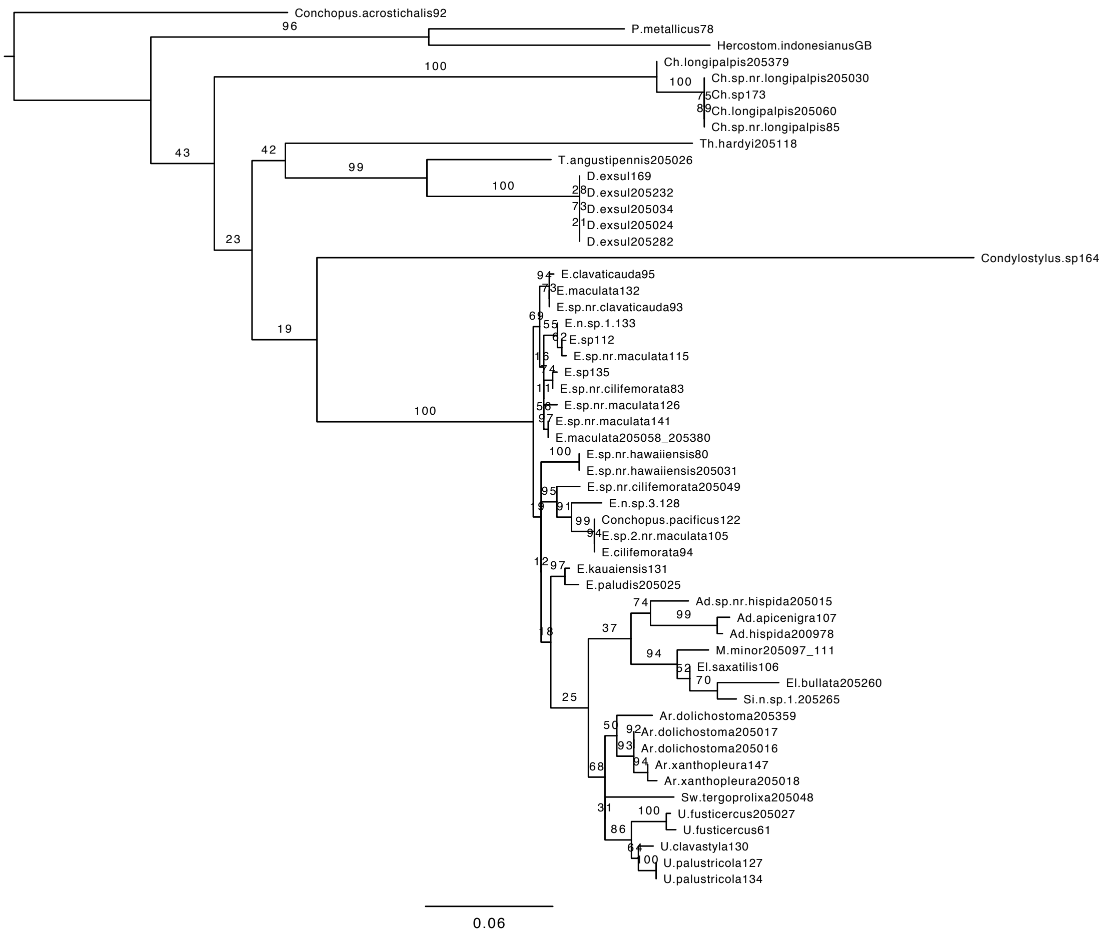

FIGURE S1I. 16S

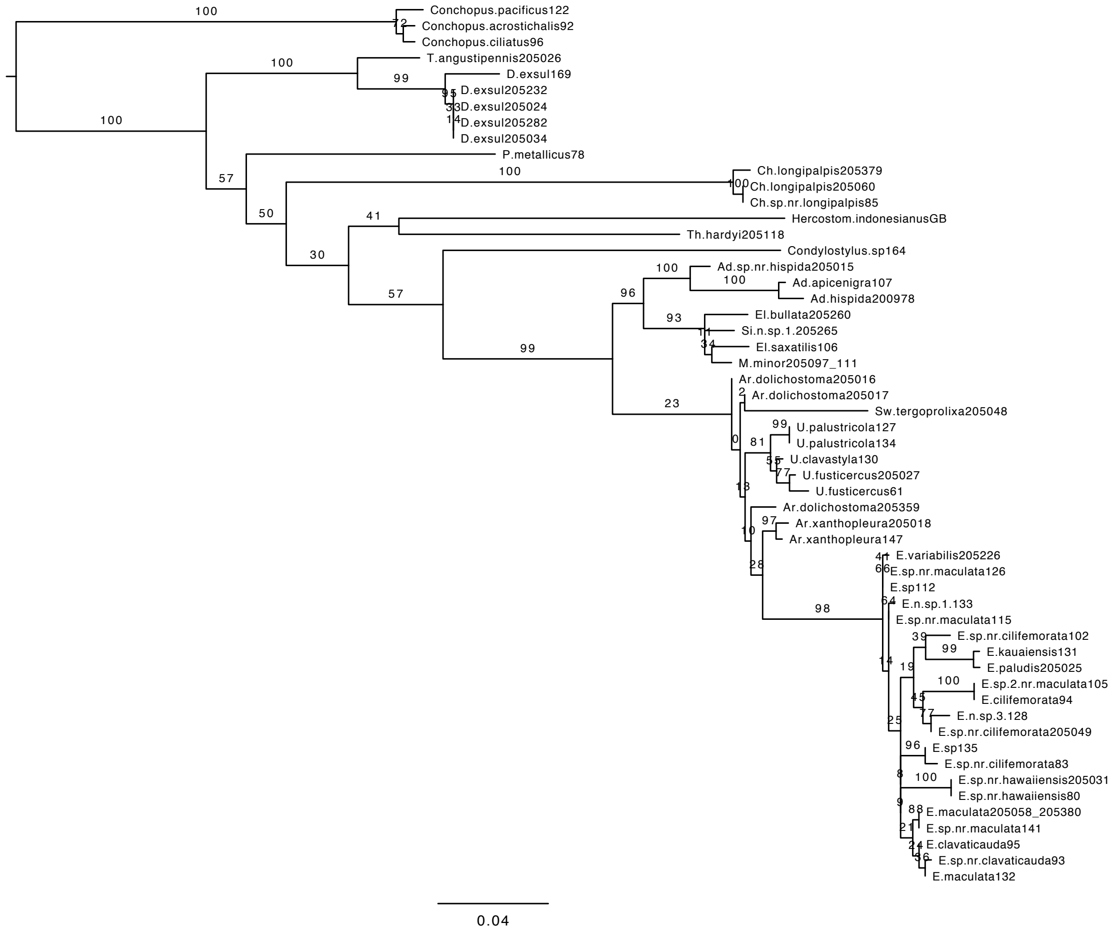

FIGURE S1J. CAD

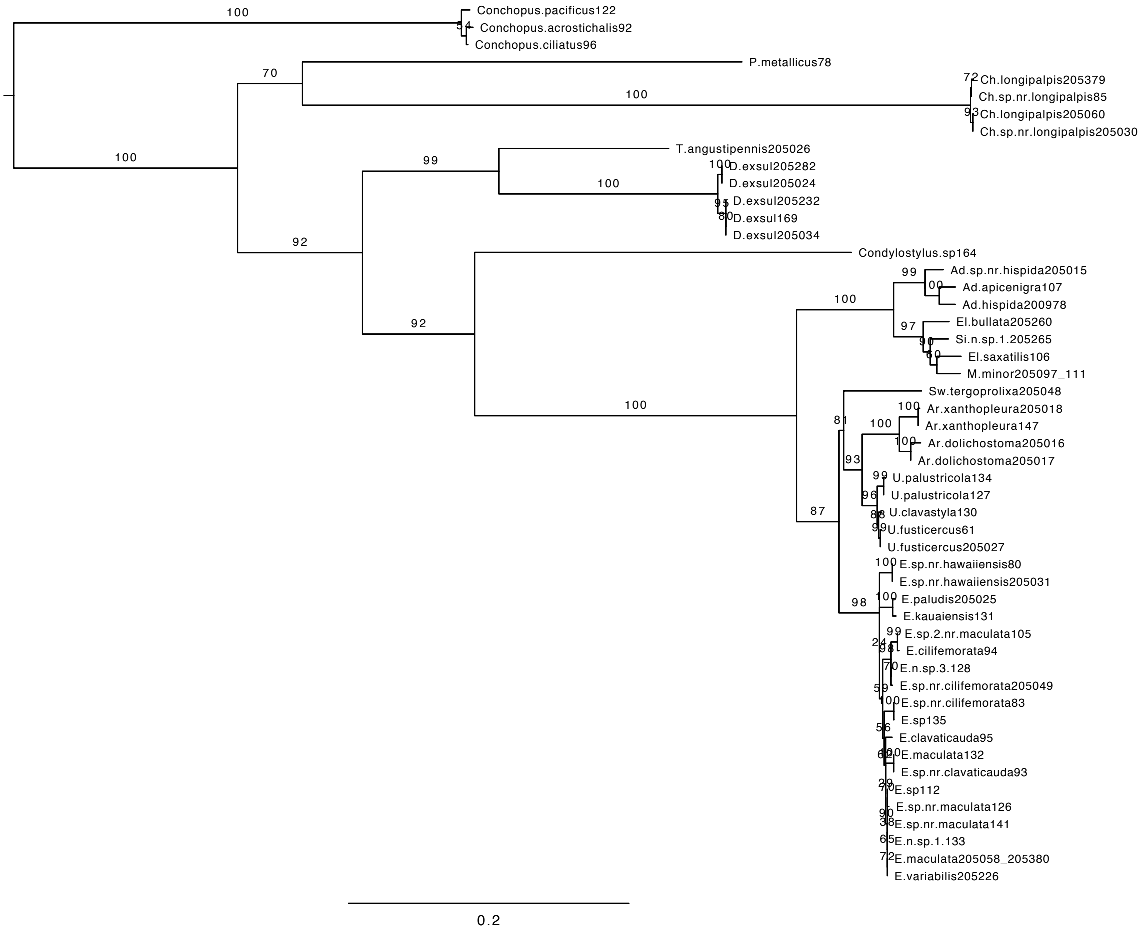

FIGURE S1K. EF1AA

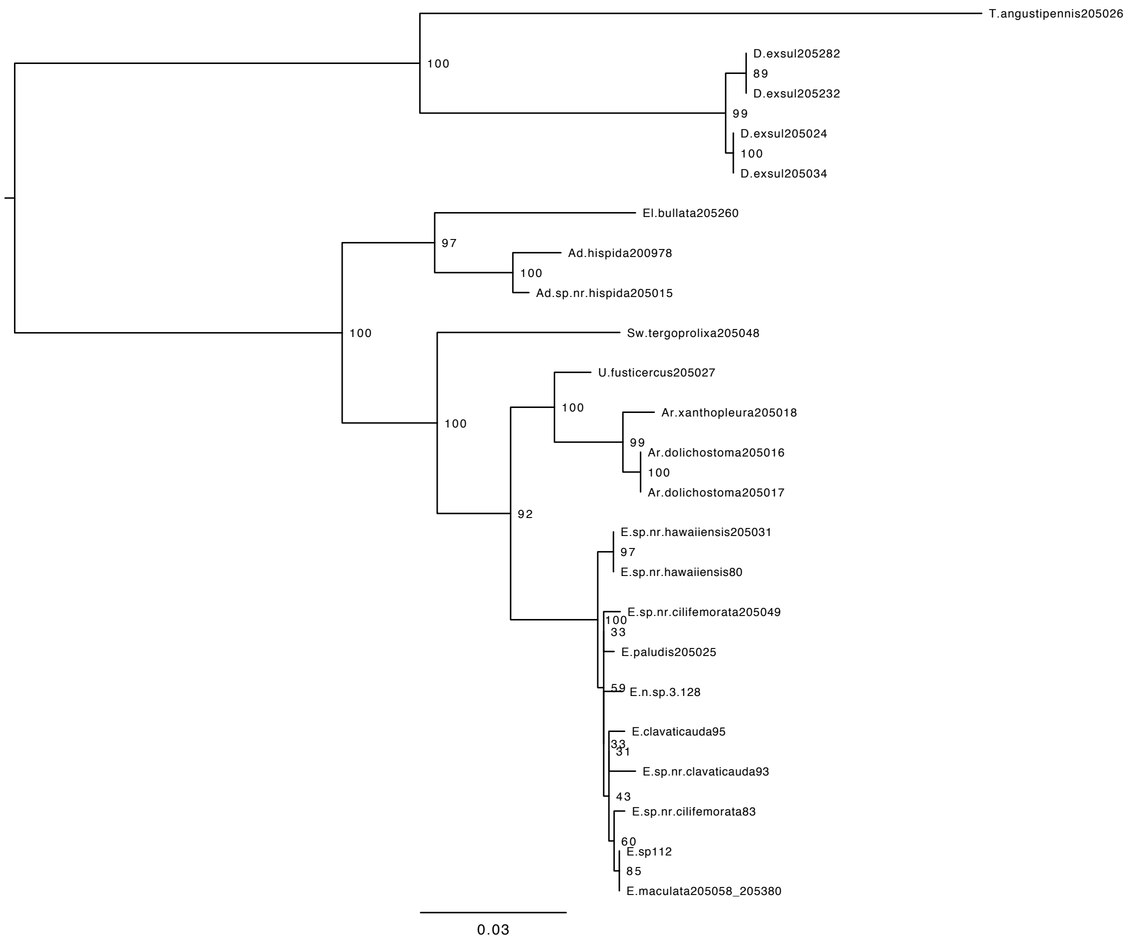

FIGURE S1L. EF1AB

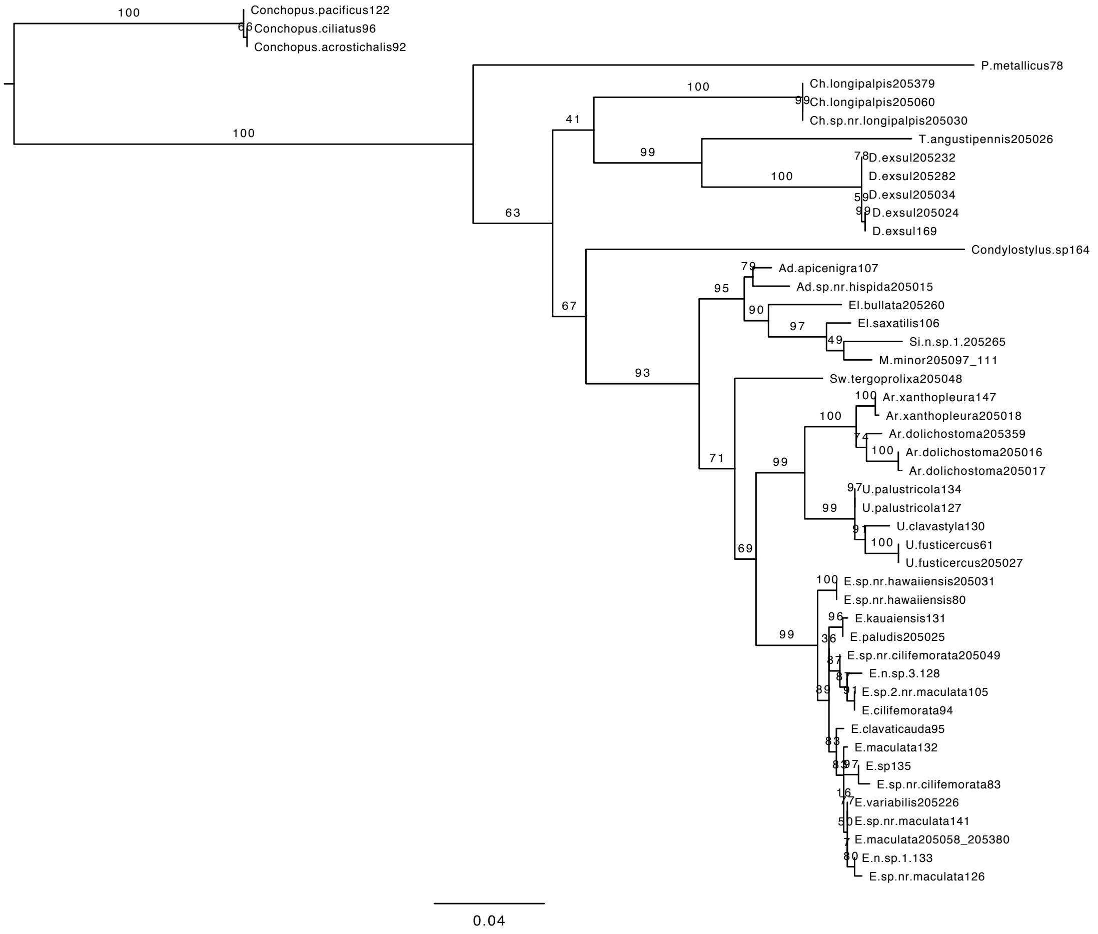

Supplement: Appendix S1 [file peerj-04-2704-s001.pdf]
