# Supplementary material for: Multiple, independent colonizations of the Hawaiian Archipelago by the family Dolichopodidae (Diptera)
Source: PeerJ. 2016 Nov 17;4:e2704. doi: 10.7717/peerj.2704 (PMC5119231; doi:10.7717/peerj.2704)
Supplement: Appendix S2 [file peerj-04-2704-s002.docx]

**Appendix S2.** Supplemental Divergence Time Estimation Analyses

**Supplemental Divergence Time Analysis Methods:**

In order to explore dating parameter space, we compared the three calibration-based analysis described in the main text to an analysis using only two calibrations (Maui and Hawaii, from Figure S2a), to two analyses using locus-specific rates. The mitochondrial gene COI has been used extensively to estimate divergence times among arthropod taxa (e.g.: (Brower, 1994; Papadopoulou *et al*., 2010; Quek *et al*., 2004)) and researchers are accustomed to thinking about molecular rates based on this gene. However, the difficulty in applying divergence rates is in selecting which rates to use, since this parameter is unknown for most species. We performed two rate-based analyses using COI rates from the literature, applying the rates as a truncated normal prior to the COI partition and allowing rates of the other partitions to vary (uniform prior: lower=1.0E-10, upper=0.5).

In the first rate-based analysis (Fast Rate), a COI divergence rate derived from Hawaiian arthropods, 5.2%/million years, was applied as a truncated normal prior to the ucld.mean parameter of the COI partition (ucld.mean: x=0.026, SD=0.007, lower=0.01 upper=0.033). This rate was based on divergence among pairs of taxa that are situated with one taxon on Maui and the other on Hawaii, and includes a wide variety of arthropods including moths, beetles, flies and spiders (data is from Table S3 within reference (Goodman *et al*., 2012)). The uncorrected pairwise sequence divergence was calculated for each pair of taxa in the table, outliers were excluded, and an average was taken. This average was divided by the age when Hawaii reached its maximum height, approximately 0.5 million years ago (Carson & Clague, 1995), which tends to be considered the most biologically plausible scenario for most taxa that rely on having mature habitats in place before being able to establish. In the second rate-based analysis (Slow Rate), a commonly applied divergence rate for the COI locus in arthropods (2.3%/million years (Brower, 1994)) was applied as a truncated normal prior to the ucld.mean parameter of the COI partition (ucld.mean: x=0.0115, SD=0.0068, lower=0.0047 upper=0.0183). The details of each analysis are presented in the main text.

**Supplemental Divergence Time Analysis Results:**

The estimated times of arrival of the *Eurynogaster* complex to the Hawaiian archipelago is similar, with overlapping confidence intervals, between the three island-calibration analyses (11.83, 95% HPD: 9.08 – 15.04, Figure S2b) Ma, the two island-calibration analysis (11.37, 95% HPD: 7.47—15.83, Figure S2c) Ma and the Fast Rate analysis 13.95 (95% HPD: 8.88-20.62) Ma (Figure S2d). The use of the Slow Rate yields a much older date of arrival to the Hawaiian archipelago, 24.56 (95%HPD: 15.88-36.10) Ma (Figure S2e). Estimates for rates of evolution at each partition are reported in Table S2b.

**Table S2a.** Nodes used for calibrating divergence time estimation in BEAST: support and calibration used.

| **Node Calibrated** | **Taxa Included** | **Support:**  **MrBayes**  **(Posterior Probability)** | **Calibration: normal distribution**  **mean (SD),**  **(95% range of time encompassed by distribution)**  (Carson and Clague, 1995) |
| --- | --- | --- | --- |
| **1. Hawaii** | E.maculata205058_205038  E.n.sp.1.133  E.sp.nr.maculata115  E.sp.nr.maculata126  E.sp.nr.maculata141  E.sp112  E.variabilis205226 | 0.97 | 0.5 (0.2)  (170 – 830 Ka) |
| **2. Maui** | E.clavaticauda95  E.maculata132  E.maculata205058_205380  E.n.sp.1.133  E.sp.nr.cilifemorata83  E.sp.nr.clavacauda93  E.sp.nr.maculata115  E.sp.nr.maculata126  E.sp.nr.maculata141  E.sp112  E.sp135  E.variabilis205226 | 1.0 | 1.3 (0.13)  (1.1 – 1.5 Ma) |
| **3. Kauai** | U.clavastyla130  U.fusticercus205027  U.fusticercus61  U.palustricola127  U.palustricola134  Ar.dolichostoma205016  Ar.dolichostoma205017  Ar.dolichostoma205359  Ar.xanthopleura147  Ar.xanthopleura205018 | 1.0 | 5.0 (0.5)  (4.2 – 5.5Ma) |

Table S2b. Comparison of results from the divergence time analyses: estimated date of arrival of the *Eurynogaster* Complex to the Hawaiian Islands and estimated evolutionary rates for each partition (ucld mean and 95% HPD).

| **Partitions:** | **Age of *Eurynogaster* Complex*,* million years**  **(95% HPD)** |  | **CADpos3** | **CADpos1, EF1αApos1,**  **EF1αBpos1** | **CADpos2**  **EF1αApos2, ND2pos2** | **CAD_Intron**  **EF1αAintron1**  **EF1αAintron2** | **EF1αApos3,**  **EF1αB pos3** | **COIpos2,**  **CO2pos2,**  **EF1αB pos2** | **EF1αB intron** | **12S,**  **16S,**  **ND2pos1** | **COIpos3,**  **CO2pos3,**  **ND2pos3** | **COIpos1,**  **CO2pos1** | |
| --- | --- | --- | --- | --- | --- | --- | --- | --- | --- | --- | --- | --- | --- |
| **3 Island Calibrations**  **(Hawaii, Maui, Kauai)** | **11.83**  **(9.08-15.04)** |  | 1.44 E-2  (1.01 E-2 – 2.05 E-2) | 1.22 E-3  (8.23 E-4 – 1.69 E-3) | 2.13 E-3  (1.32 E-3 – 3.30 E-3) | 1.66 E-2  (1.09 E-2 –  2.37 E-2) | 5.96 E-3  (4.40 E-3 –  7.80 E-3) | 4.78 E-4  (2.77 E-4 – 7.35 E-4) | 4.58 E-2  (1.99 E-2 – 0.11) | 9.77 E-3  (7.01 E-3 – 1.32 E-2) | 7.51 E-2  (5.74 E-2 – 9.78 E-2) | 1.16 E-2  (8.26 E-3 – 1.56 E-2) | |
| **2 Island Calibrations**  **(Hawaii, Maui)** | **11.37**  **(7.47-15.83)** |  | 1.58 E-2 (9.75 E-3 – 2.38 E-2) | 1.30 E-3  ( 8.25 E-4 – 1.92 E-3) | 2.08 E-3  (1.25 E-3 – 3.28 E-3) | 1.72 E-2  (1.04 E-2 – 2.59 E-2) | 6.41 E-3  (4.15 E-3 – 9.13 E-3) | 5.23 E-4  (2.79 E-4 – 8.65 E-4) | 6.09 E-2 (1.99 E-2 – 0.13) | 9.53 E-3 (6.35 E-3 – 1.38 E-2) | 7.95 E-2 (5.36 E-2 – 0.11) | 1.2 E-2 (7.53 -3 – 1.77 E-2) | |
|  |  |  |  |  |  |  |  |  |  |  |  |  | |
| **Evolutionary Rates** |  | **COI** | **CADpos3** | **CADpos1, EF1αApos2,**  **EF1αBpos1** | **CADpos2**  **EF1αApos2, ND2pos2** | **CAD_Intron**  **EF1αAintron1**  **EF1αAintron2** | **EF1αApos3,**  **EF1αB pos3** | **CO2pos2,**  **EF1αB pos2** | **EF1αB intron** | **12S,**  **16S,**  **ND2pos1** | **CO2pos3,**  **ND2pos3** | **CO2pos1** |  |
| **Slow Rate (2.3%)** | **24.56**  **(15.88-36.10)** | 1.66 E-2  (1.37 E-2 – 1.83 E-2) | 6.87 E-3  (3.89 E-3 – 1.10 E-2) | 5.64 E-4  (3.22 E-4 – 8.59 E-4) | 9.59 E-4  (5.20 E-4 – 1.56 E-3) | 7.71 E-3  (4.26 E-3 –  1.21 E-2) | 2.76 E-3  (1.68 E-3 –  4.02 E-3) | 1.94 E-4  (9.32 E-5 – 3.25 E-4) | 2.15 E-2  (7.97 E-3 – 5.76 E-2) | 4.23 E-3  (2.53 E-3 – 6.04 E-3) | 2.97 E-2  (1.82 E-2 – 4.27 E-2) | 2.87 E-3 (1.75 E-3 – 4.19 E-3) |  |
| **Fast Rate (5.2%)** | **13.95**  **(8.88-20.62)** | 3.06 E-2  (2.49 E-2 – 3.3 E-2) | 1.22 E-2  (6.76 E-3–  1.89 E-2) | 9.97 E-4  (5.73 E-4 –  1.52 E-3) | 1.70 E-3  (8.91 E-4-  2.76 E-3) | 1.34 E-2  (7.42 E-3 –  2.10 E-2) | 4.87 E-3  (2.95 E-3 –  7.13 E-3) | 3.42 E-4  (1.61 E-4 – 5.74 E-4) | 3.70  (1.34 E-2 – 9.43 E -2) | 7.35 E-3 (4.47 E-3 – 1.1 E-2) | 5.23 E-2  (3.18 E-2 – 7.79 E-2) | 5.08 E-3  (3.02 E-3 – 7.44 E-3) |  |

**Figure S2a.** Calibrations used in the divergence time analysis (described in table S2a)

**
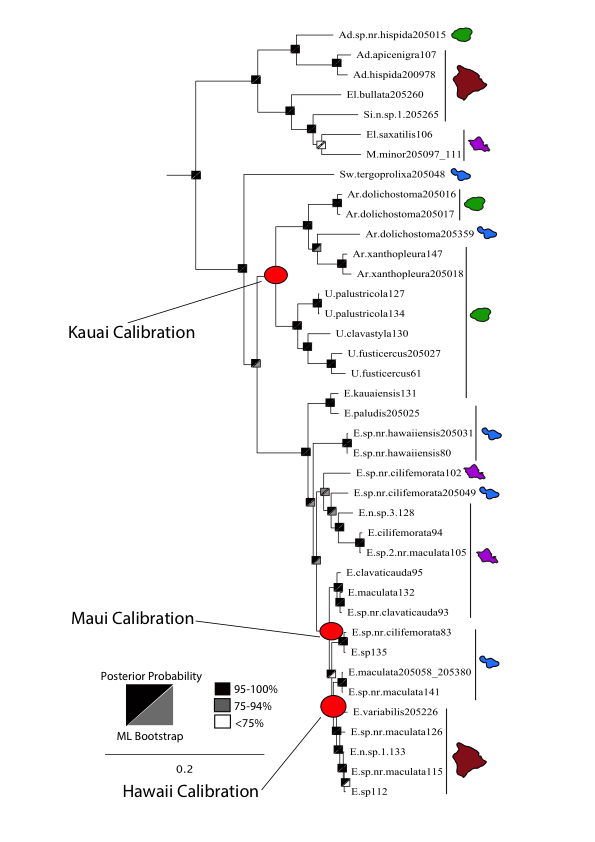
**

**Figure S2b.** Beast 3 calibration time tree (Kauai, Maui, Hawaii ; Table S2a, Figure S2a). **
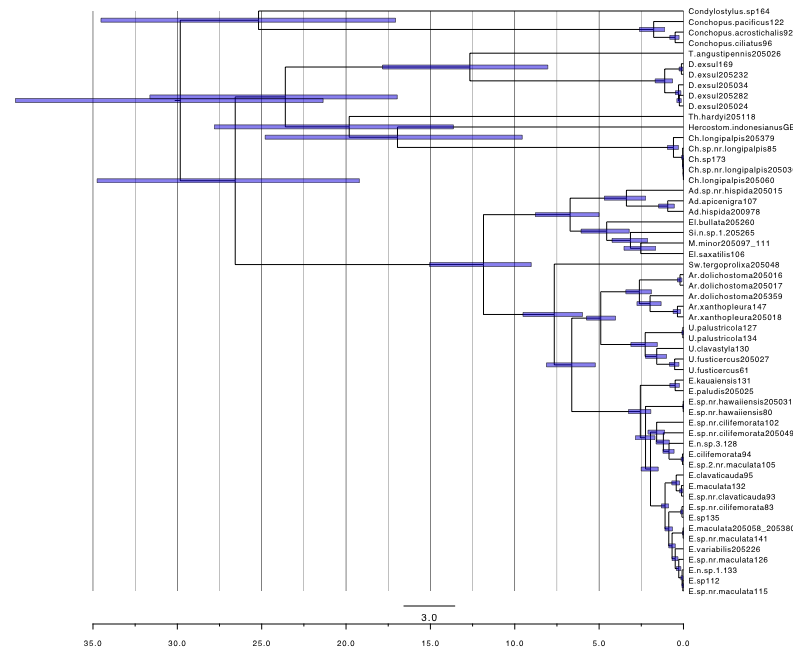
**

**Figure S2c.** Beast 2 calibration time tree (Maui, Hawaii ; Table S2a, Figure S2a). **
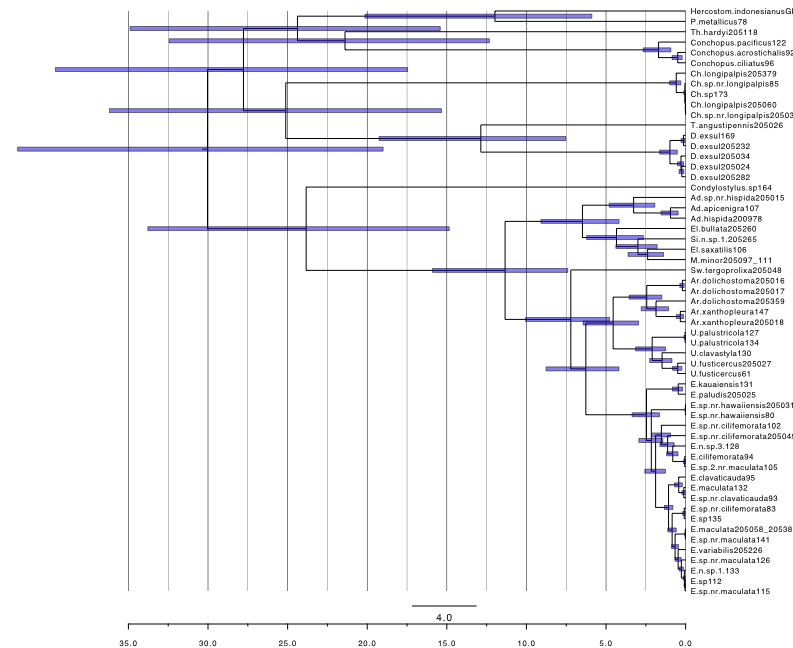
Figure S2d.** Alternate BEAST Tree using COI divergence rate, Fast Rate analysis (5.2%/million years):**
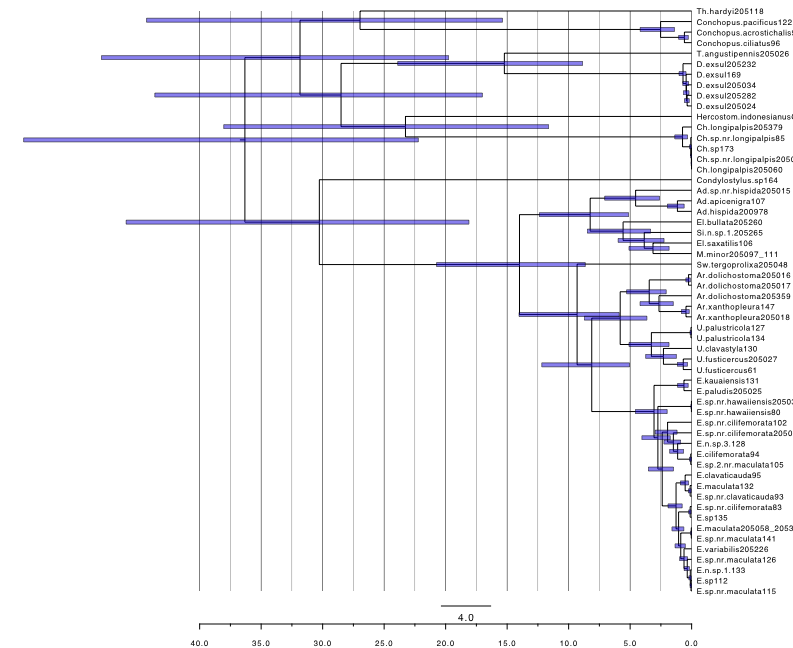
**

**Figure S2e.** Alternate BEAST Tree using COI divergence rate, Slow rate analysis (2.3%/million years):
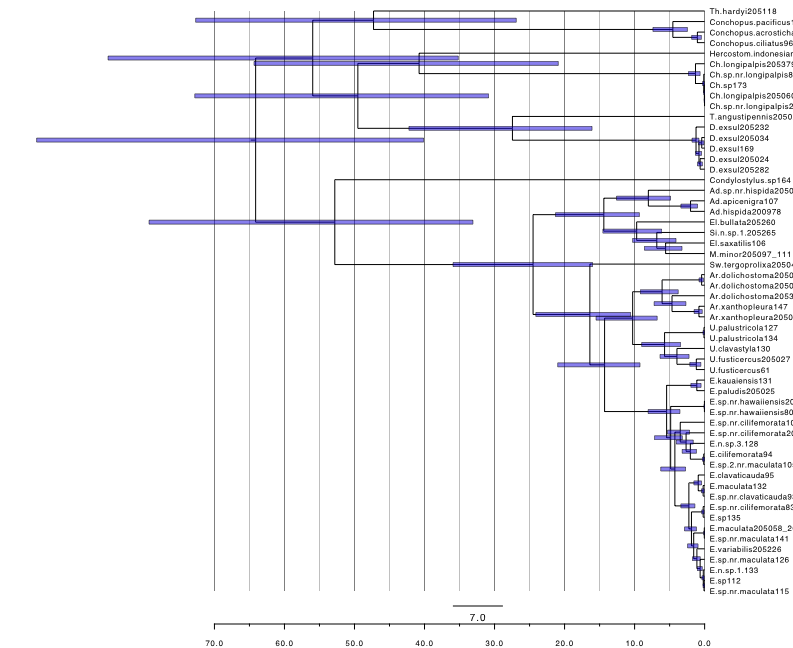


**REFERENCES**

Brower, A.V.Z. (1994) Rapid morphological radiation and convergence among races of the butterfly *Heliconius erato* inferred from patterns of mitochondrial DNA evolution. *Proceedings of the National Academy of Sciences*, **91**, 6491-6495.

Carson, H.L., Clague, D.A. (1995) Geology and biogeography of the Hawaiian Islands. In: Wagner, W.L., Funk, V.A. (Eds.), *Hawaiian biogeography: evolution on a hotspot archipelago*. Smithsonian Institution Press, Washington, D.C., pp. 14-29.

Goodman, K.R., Welter, S.C., Roderick, G.K. (2012) Genetic divergence is decoupled from ecological diversification in the Hawaiian *Nesosydne* planthoppers. *Evolution*, **66**, 2798-2813. .

Papadopoulou, A., Anastasiou, I., Vogler, A.P. (2010) Revisiting the insect mitochondrial molecular clock: the mid-Aegean trench calibration. *Molecular Biology and Evolution*, **27**, 1659-1672.

Quek, S.P., Davies, S.J., Itino, T., Pierce, N.E. (2004) Codiversification in an ant-plant mutualism: Stem texture and the evolution of host use in Crematogaster (Formicidae : Myrmicinae) inhabitants of Macaranga (Euphorbiaceae). *Evolution,* **58**, 554-570.
