# Supplementary material for: Multiple, independent colonizations of the Hawaiian Archipelago by the family Dolichopodidae (Diptera)
Source: PeerJ. 2016 Nov 17;4:e2704. doi: 10.7717/peerj.2704 (PMC5119231; doi:10.7717/peerj.2704)
Supplement: Appendix S3 [file peerj-04-2704-s003.pdf]

Appendix S3. History of taxonomy of the *Eurynogaster* complex

The genus *Eurynogaster* (subfamily Sympycninae) was originally proposed by Van Duzee (1933) based on three species he described, two from Oahu and one from Hawaii Island (Figure S3a). In the same paper, he also proposed the genus *Sweziella* and described a single species from Oahu (*S. albifacies*), placing this genus in the subfamily Thinophilinae (Figure S3a). Parent (1934) proposed the genus *Pachyurus* for the single endemic Hawaiian species, *P. hawaiensis* (Figure S3a). Later, noting the name was preoccupied, Parent (1935) proposed *Uropachys* as a replacement name and placed the genus in the subfamily Hydrophorinae (Figure S3a). Parent (1940) synonymized *Sweziella* under *Eurynogaster* based on the advice of F.X. Williams (*in litt*.). Hardy & Kohn (1964) and Tenorio (1969), in their respective revisionary works on the dolichopodids of Hawaii, treated both *Sweziella* and *Uropachys* as junior synonyms under *Eurynogaster* (Figure S3a) based primarily on the shared presence of a prescutellar depression. Evenhuis (2005) noted that the prescutellar depression was merely an artifact of air drying and not of any phylogenetic importance. Furthermore, he examined the thoracic and leg characters and male genitalia and concluded that the species previously assigned to *Eurynogaster sensu* Hardy (1964) and *sensu* Tenorio (1969) actually belonged to a complex of genera (Figure S3a). In the same study, Evenhuis (2005) re-examined the closely related endemic Hawaiian genus *Sigmatineurum* Parent and found that it also belonged in the *Eurynogaster* complex of genera. The close association of *Sigmatineurum* and *Eurynogaster* was further supported the transfer of several *Eurynogaster* species to the genus *Sigmatineurum* (Evenhuis 2005). In summary (Figure S3a), Evenhuis (2005) resurrected *Sweziella* and *Uropachys* from *Eurynogaster,* included

Sigmatineurum, and described four new genera (*Adachia, Arciellia, Elmoia,* and *Major*) to accommodate all the species previously lumped in *Eurynogaster* by (Hardy & Kohn, 1964) and Tenorio (1969).

Figure S3a. Taxonomic history of the *Eurynogaster* Complex. Convergent and divergent lines track dates of synonymy and description of new genera, respectively. Dots show year and number of species described. Color of dot indicates which present-day genus the species is placed within. Grimshaw (1901) described three species which predate the currently recognized genera; two are currently placed in *Eurynogaster* and one is in *Elmoia*.

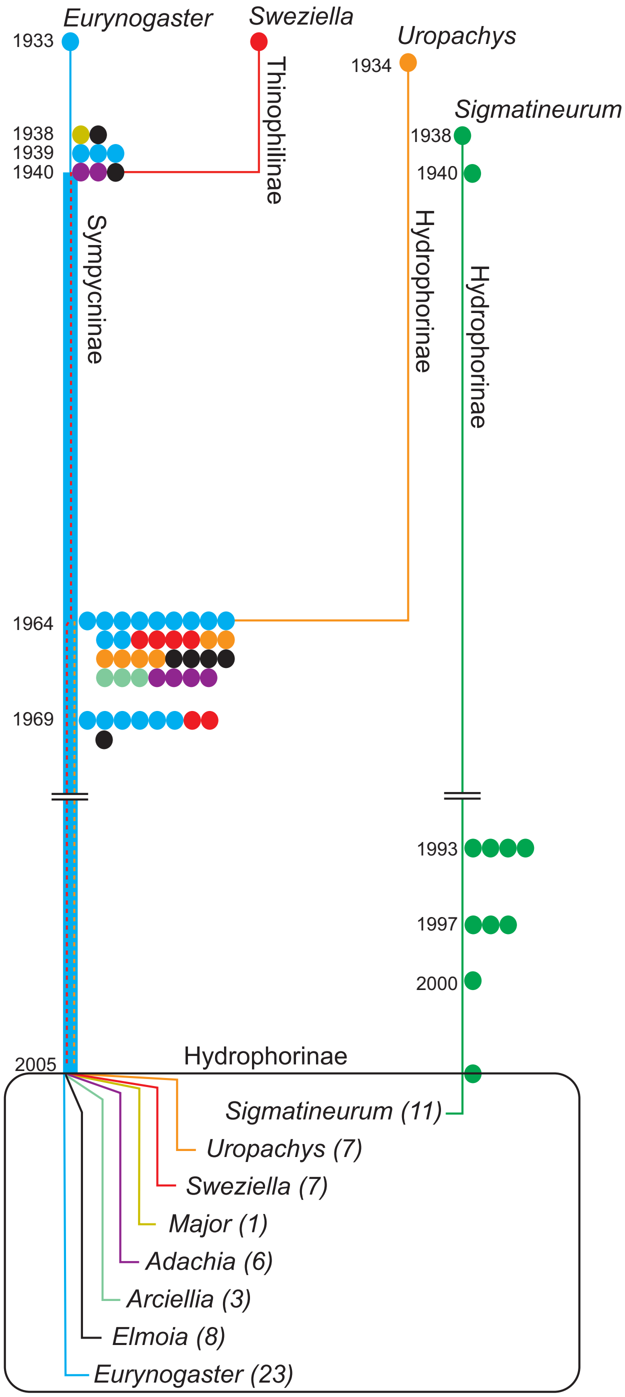

REFERENCES

Evenhuis, N.L. (2005) A review of the genera comprising species of the genus Eurynogaster sensu Hardy & Kohn, 1964 in Hawaii (Diptera: Dolichopodidae). *Zootaxa*, **1017**, 39-60.

Hardy, D.E. (1964) Diptera: Brachycera, Family Dolichopodidae, Cyclorrhapha, series Aschiza, Families Lonchopteridae, Phoridae, Pipunculidae, and Syrphidae. *Insects of Hawaii*, **11**, 458.

Hardy, D.E. & Kohn, M.A. (1964) Dolichopodidae. *Insects of Hawaii*, **11**, 1–256.

Parent, O. (1934) Diptères dolichopodides exotiques. *Mémoires de la Société Nationale des Sciences Naturelles et Mathématiques de Cherbourg*, **41**, 257-308.

Parent, O. (1940) Dolichopodides des Iles Hawaiʻi recueillis par Monsieur F.W. Williams, principalement au cours de l'année 1936. *Proceedings of the Hawaiian Entomological Society*, **10**, 225-249.

Tenorio, J.M. (1969) Supplement, Diptera: Dolichopodidae, Appendix (Phoridae). *Insects of Hawaii, Vol. 11*, p. v + 73. University of Hawaii Press, Honolulu, HI.

Van Duzee, E.C. (1933) New Dolichopodidae from the Hawaiian Islands (Diptera). *Proceedings of the Hawaiian Entomological Society*, **8**, 309-356.
