# Supplementary material for: Multiple, independent colonizations of the Hawaiian Archipelago by the family Dolichopodidae (Diptera)
Source: PeerJ. 2016 Nov 17;4:e2704. doi: 10.7717/peerj.2704 (PMC5119231; doi:10.7717/peerj.2704)
Supplement: Appendix S4 [file peerj-04-2704-s004.pdf]

7 September 2011

Mark Vaught
East Maui Irrigation Company
(808) 579-9516

Dear Mark,

We are interested in applying for access to the Waikamoi Forest Preserve for 22 and 23 September 2011. The purpose of my visit is to collect native Hawaiian insects. The specimens obtained during this trip will be returned to the University of California Berkeley for identification. Some material will be placed in the B.P. Bishop Museum and other specimens will be used for DNA extraction and analysis.

**Trip Itinerary**

***Arrival:*** 21 September 2011, Hawaiian Airlines #374 at 3:19pm, transfer to Hawaiian Airlines #544 in Honolulu at 4:23pm, arrive Kahului at 5pm
***Departure:*** 24 September 2011, Hawaiian Airlines #543 at 5:28pm, transfer to Hawaiian Airlines #362 in Honolulu at 6:23pm, arrive Hilo at 7:23pm

We plan to be in the field 8am-4pm on the 22$^{nd}$ and 23$^{rd}$.

**Contact information:**

Dr. Patrick O'Grady, Associate Professor, Department of Environmental Science, Policy and Management, University of California, Berkeley, CA 94720, email: ogrady@berkeley.edu, cell: (802) 370-9066

Dr. Brian Ort, Postdoctoral Researcher, Department of Environmental Science, Policy and Management, University of California, Berkeley, CA 94720, email: ort@berkeley.edu, cell: 541-602-0454

Dr. Kari Goodman, Postdoctoral Researcher, Department of Environmental Science, Policy and Management, University of California, Berkeley, CA 94720, email: krgoodman@berkeley.edu, cell 510-913-2109

Heather Machado, Graduate Student, Department of Ecology and Evolution, Stanford University, Palo Alto, CA email: hmachado@stanford.edu, cell: 503-701-6356.

Thank you for your assistance. If you require further information, please contact me at (802) 656-0460 or via email: ogrady@berkeley.edu

Sincerely,

Patrick O'Grady

# APPLICATION FOR A SCIENTIFIC RESEARCH AND COLLECTING PERMIT

United States Department of the Interior

National Park Service

All or some of the information you provide may become available to the public.

OMB # (1024-0236)
Exp. Date (02/28/2014)
Form No. (10-741a)

**Name of the National Park Service area you are applying to:** Hawaii Volcanoes NP

| **Type of application:** | **Please enter numbers for permit renewal or modification requests:** |
|---|---|
| Renewal application | **Previously assigned NPS study number:** HAVO-00312<br>**Previously assigned NPS permit number:** HAVO-2011-SCI-0010 |

**Contact information for the current principal investigator**

| | |
|---|---|
| **Principal investigator:**<br>Patrick O'Grady | **Office phone:**<br>(510) 642-0662 |
| **Mailing address of principal investigator:**<br>University of California, Berkeley<br>Department of Environmental Science, Policy & Management<br>Division of Organisms and the Environment<br>137 Mulford Hall<br>Berkeley, CA 94720<br>United States | **Alternative phone:**<br>(510) 642-0662<br>**Office fax:**<br>(510) 643-5098 |
| **Name of the current institution represented**<br>University of California, Berkeley | **Office email address:**<br>ogrady@drosophilaevolution.com |

**Additional investigators or key field assistants (first name, last name, office phone, office email)**

| | | |
|---|---|---|
| **Name:** jessica craft | **Phone:** 7605203144 | **Email:** jcraft86@berkeley.com |
| **Name:** Gordon Bennett | **Phone:** | **Email:** |
| **Name:** brian ort | **Phone:** | **Email:** ort@berkeley.edu |
| **Name:** kari roesch goodman | **Phone:** | **Email:** |
| **Name:** Gordon Bennett | **Phone:** | **Email:** |
| **Name:** lisa marrack | **Phone:** | **Email:** |

**Scientific Study Information**

**Project title (maximum 300 characters)**

A Comparative Approach to the Evolutionary Biology of Hawaiian Insects: Population Genetic and Phylogenetic Studies

**Purpose of the study (maximum 4000 characters)**

Since the early 1960â s Hawaiian Drosophila have been used as a model system to understand the mechanics of the evolutionary process. Their biogeography is remarkable; it is an example of rapid adaptive radiation on one of the most isolated island systems in the world. The seclusion of this lineage from mainland populations makes it a particular system of importance for further study and comparative analyses. Our research focuses on synthesizing and expanding on the accumulated research from decades of study on Hawaiian Drosophila by (1) using phylogenetic approaches to comparatively examine different lineages of endemic Hawaiian insects and (2) focusing on the genetic mechanisms of speciation at the population level.

Examining the pattern, timing, and rate of diversification in other Hawaiian insect groups (Nesophrosyne leaf hoppers and limoniid craneflies) allows us to infer common evolutionary processes. As an example, a set molecular clock can be compared to phylogenetic

hypotheses of independent Hawaiian lineages to infer information about the time of colonization and rates of speciation. Biogeographic patterns can also allow us to infer how intra-island colonization and subsequent speciation events have proceeded down the island chain. A population genetics approach will also be useful in understanding how species form, particular within large, rapidly evolving lineages.

We will examine seven species of the endemic spoon tarsus clade within Hawaiian Drosophila for this study. At less than 500,000 years old, this group is recent compared to other clades of Hawaiian Drosophila. The fact that several species seemed to have formed in the past half million years, implies the speciation is either recent or ongoing in this group. By observing the evolutionary forces acting on these populations, we can gain a better understanding of the effect of gene flow, hybridization, and ancestral population size on the diversification process.

**Summary of proposed field methods and activities (extract from the study proposal where appropriate - maximum 4000 characters)**

| Study Schedule | Field Schedule |
|---|---|
| **Initial starting date of the study:**<br>Jun 01, 2009<br>**Estimated date the entire study may end:**<br>Jul 01, 2014 | **Date to begin study within the park this application year:**<br>Jun 11, 2012<br>**Date to end study within the park this application year:**<br>Jun 11, 2013<br>**Will field study need to continue within the park next year:**<br>Yes |

**Activity Type:** Research

**Do you anticipate receiving funding assistance from the U.S. Federal Government for this study? (Yes or No)**

Yes

**If yes specify the agency(s):**

National Science Foundation

**Where will data, maps, photos, etc. (not specimens) reside upon completion of this study?**

**Location(s) where you propose activities will take place within the National Park System area(s):**

Projected collections will occur at mesic-dry forest habitats and rain forest habitats. Some of these areas include Kipuka puaulu, Muana Loa raod and lookout, Ola'a forest, Escape Road, Napau trail forests, and Pu'u Hululu. Collections may extend beyond these points within the park.

**Your proposed method of access (vehicles, aircraft, boat, snowmobile, foot, etc.):**

We will access the park via motor vehicle. Collections will be made on foot.

# APPLICATION FOR A SCIENTIFIC RESEARCH AND COLLECTING PERMIT

United States Department of the Interior

National Park Service

All or some of the information you provide may become available to the public.

OMB # (1024-0236)
Exp. Date (02/28/2014)
Form No. (10-741a)

**Handling or Collection of Specimens**

**Would you like to handle or collect specimens?** Yes

If you respond "Yes", please complete this entire section of the application (otherwise you may skip the remainder of this section).

**Scientific description of specimens to be handled or collected (include taxonomic group or name, or type of material; sample size, quantity, frequency, and location):**

Scaptomyza abrupta Maui 15

Scaptomyza affinicuspidata Maui 15

Scaptomyza abberans Molokai 15

Scaptomyza buccata Hawaii, Maui, Molokai, Oahu 15

Scaptomyza cerina Molokai 15

Scaptomyza fusciforns Oahu, Kauai 15

Scaptomyza longisetosa Hawaii, Maui, Molokai 15

Scaptomyza mutica Maui 15

Scaptomyza semiflavs Maui, Molokai, Oahu 15

Scaptomyza stramineifrons Oahu 15

Scaptomyza anomala Maui, Kauai 15

Scaptomyza bryanti Hawaii, Maui 15

Scaptomyza cnecosoma Oahu 15

Scaptomyza confusa Maui 15

Scaptomyza hamata Kauai 15

Scaptomyza palmae Hawaii, Maui, Oahu 15

Scaptomyza varifrons Oahu 15

Scaptomyza mauiensis Hawaii, Maui, Molokai, Oahu, Kauai 15

Scaptomyza pallida Hawaii, Maui, Molokai, Oahu, Kauai 15

Scaptomyza aloha Molokai 15

Scaptomyza finitima Oahu 15

Scaptomyza mediopallens Hawaii 15

Scaptomyza mitchelli Hawaii 15

Scaptomyza striatifrons Kauai 15

Scaptomyza albovittata Oahu 15

Scaptomyza brunnimaculata Molokai 15

Scaptomyza flavida Molokai 15

Scaptomyza gilvivirilia Maui 15

Scaptomyza nigrosignata Hawaii 15

Scaptomyza varipicta Hawaii 15

Scaptomyza acronastes Oahu 15

Scaptomyza adunca Oahu 15

Scaptomyza affinicuspidata Maui 15

Scaptomyza anechocerca Kauai 15

Scaptomyza apiciguttula Hawaii 15

Scaptomyza apponopusilla Hawaii 15

Scaptomyza agrentifrons Kauai 15

Scaptomyza articulata Hawaii, Molokai 15

Scaptomyza basiloba Hawaii 15

Scaptomyza bilobata Kauai 15

Scaptomyza bipars Kauai 15

Scaptomyza brachycerca Hawaii 15

Scaptomyza camptochaites Maui 15

Scaptomyza concinna Maui 15

Scaptomyza connata Hawaii 15

Scaptomyza cornuta Hawaii 15

Scaptomyza cryptoloba Maui. Oahu 15

Scaptomyza ctenophora Maui 15

Scaptomyza cuspidata Hawaii, Maui 15

Scaptomyza decepta Kauai 15

Scaptomyza denata Kauai 15

Scaptomyza devexa Maui 15

Scaptomyza diaphorocerca Molokai 15

Scaptomyza domita Maui 15

**Scaptomyza dubautiae Maui 15**

**Scaptomyza dubia Molokai, Kauai 15**

Scaptomyza eurystylata Hawaii, Maui, Oahu, Kauai 15

Scaptomyza evexa Kauai 15

Scaptomyza exigua Hawaii, Maui 15

Scaptomyza fastigata Hawaii, Maui, Molokai 15

Scaptomyza hacmani Hawaii, Maui, Molokai 15

Scaptomyza hardyi Kauai 15

Scaptomyza inaequalis Hawaii, Maui, Molokai 15

Scaptomyza inermis Hawaii 15

Scaptomyza infurcula Hawaii 15

Scaptomyza innotabilis Kauai 15

Scaptomyza intricata Maui, Molokai 15

Scaptomyza isopedon Hawaii, Maui 15

Scaptomyza kauaiensis Kauai 15

Scaptomyza latitergum Maui 15

Scaptomyza levata Hawaii, Oahu, Kauai 15

Scaptomyza lobifera Hawaii 15

Scaptomyza longipecten Maui, Molokai 15

Scaptomyza mecocerca Kauai 15

Scaptomyza mediana Maui 15

Scaptomyza mimula Molokai 15

Scaptomyza monticola Hawaii 15

Scaptomyza multidenta Hawaii 15

Scaptomyza obscuricornia Maui, Molokai, Oahu 15

Scaptomyza ochromata Maui 15

Scaptomyza ostensa Maui 15

Scaptomyza pallifrons Hawaii, Maui, Molokai, Kauai 15

Scaptomyza paralobe Hawaii 15

Scaptomyza penicula Kauai 15

Scaptomyza photophilia Hawaii 15

Scaptomyza phyrysothrix Molokai 15

Scaptomyza protensa Molokai 15

Scaptomyza punctivena Maui 15

Scaptomyza pusilla Hawaii, Maui, Molokai 15

Scaptomyza quadridentata Oahu 15

Scaptomyza recava Hawaii 15

Scaptomyza recta Maui 15

Scaptomyza retusa Molokai 15

Scaptomyza robusta Maui 15

Scaptomyza rotundiloba Oahu 15

Scaptomyza scoliops Oahu 15

Scaptomyza scoloplichas Hawaii 15

Scaptomyza setiger Hawaii 15

Scaptomyza setosiloba Hawaii 15

Scaptomyza silcivola Hawaii, Maui, Molokai 15

Scaptomyza spilota Maui 15

Scaptomyza tenuata Hawaii 15

Scaptomyza trivatata Kauai 15

Scaptomyza tumidula Hawaii 15

Scaptomyza uliginosa Hawaii, Maui 15

Scaptomyza umrbrosa Oahu, Kauai 15

Scaptomyza univitta Kauai 15

Scaptomy vagabunda Oahu, Kauai 15

Scaptomyza varia Hawaii, Maui, Molokai 15

Scaptomyza villosa Kauai 15

Scaptomyza waialeaeleae Kauai 15

**Proposed disposition of specimens identified for handling or collection:**

Temporarily retained, then destroyed through analysis or discarded after analysis (If your analysis later determines that temporarily retained specimens or material originating from such specimens in fact warrant permanent retention, contact the park research coordinator for further instructions)

**If you propose that specimens and/or material originating from such specimens be retained permanently, they will become part of National Park Service collections. You may request that such specimens and/or material originating from such specimens be loaned to one or more non-NPS institutions for management. If you do make this request, you accept responsibility for obtaining and submitting to NPS the signature of the official at each proposed repository using the form attached as Appendix A. Note that all specimens collected and material originating from such specimens remain Federal property.**

**Certification**

**I certify that this application is accurate and complete. I understand a formal study (research) proposal for new or modified studies must be provided to NPS before this application can be considered. I authorize the National Park Service to seek peer reviews of my proposal.**

Signature of principal investigator: [signature] Date: 27 July 2012

| For National Park Service use only | Date received | Assigned study number | Assigned permit number |
|---|---|---|---|
| | | | |

**Paperwork Reduction Act Statement:** A federal agency may not conduct or sponsor, and a person is not required to respond to a collection of information unless it displays a valid OMB control number. Public reporting for this collection of information form is estimated to average 1.38 hours per response, including the time for reviewing instructions, gathering and maintaining data, and completing and reviewing the forms. Direct comments regarding this burden estimate or any aspect of this form to Dr. John G. Dennis, Natural Resources (3130 MIB), National Park Service, 1849 C Street, N.W., Washington, DC 20240.
**Privacy Act Notice:** Scientific research, education and collecting activities within units of the National Park System that may impact parks invoke a permitting and reporting requirement per regulations at 36 CFR 1.6 (Permits), 36 CFR 2.1 (Preservation of Natural, Cultural and Archeological Resources), and 36 CFR 2.5 (Research Specimens). The National Park Service collects information about permit applicants and permittees to administer and document research, collecting, and reporting activities within parks. The information disclosed on this form is required and may result in denial of permit applications if not provided.

# APPLICATION FOR A SCIENTIFIC RESEARCH AND COLLECTING PERMIT

United States Department of the Interior

National Park Service

All or some of the information you provide may become available to the public.

OMB # (1024-0236)
Exp. Date (02/28/2014)
Form No. (10-741a)

**Handling or Collection of Specimens**

**Would you like to handle or collect specimens?** Yes

If you respond "Yes", please complete this entire section of the application (otherwise you may skip the remainder of this section).

**Scientific description of specimens to be handled or collected (include taxonomic group or name, or type of material; sample size, quantity, frequency, and location):**

Drosophila waddingtoni Hawaii, Maui Nui 70

Drosophila dasycnemia Hawaii 30

Drosophila septuosa Hawaii 30

Drosophila sordidapex Hawaii 30

Drosophila percnosoma Hawaii 30

Drosophila neutralis Hawaii 30

Drosophila incognita Hawaii 30

Drosophila conformis Hawaii 30

Drosophila mimica Hawaii 15

Drosophila cneucopleura Hawaii 15

Scaptomyza cyrtandrae Hawaii 15

Drosophila imparisetae Hawaii 15

Nesophrosyne affinis Hawaii 10

Nesophrosyne craterigena Hawaii 10

Nesophrosyne ehu Hawaii, Maui 10

Nesophrosyne giffardi Hawaii, Lanai, Oahu 10

Nesophrosyne giffardi interrupta Hawaii 10

Nesophrosyne ignigena Hawaii 10

Nesophrosyne imbricola Hawaii, Lanai 10

Nesophrosyne mabae Hawaii 10

Nesophrosyne montium Hawaii 10

Nesophrosyne montivaga Hawaii 10

Nesophrosyne myrsines Hawaii 10

Nesophrosyne nuenue Hawaii, Lanai 10

Nesophrosyne oreadi Hawaii, Oahu 10

Nesophrosyne pele Hawaii 10

Nesophrosyne pipturi Oahu, Molokai, Lanai, Maui 10

Nesophrosyne pluvialis Hawaii, Oahu 10

Nesophrosyne silvicola Hawaii, Lanai 10

Nesophrosyne sinuate Hawaii, Oahu 10

Nesophrosyne touchardii Hawaii, Oahu 10

Nesophrosyne ulaula Hawaii, Oahu, Maui 10

Campsicnemus brevitibia Hawaii 5

Campsicnemus crassipes Hawaii 5

Campsicnemus dicondylus Hawaii 5

Campsicnemus setiger Hawaii 5

Campsicnemus loxothrix Hawaii 5

Campsicnemus flavipes Hawaii 5

**Proposed disposition of specimens identified for handling or collection:**

Temporarily retained, then destroyed through analysis or discarded after analysis (If your analysis later determines that temporarily retained specimens or material originating from such specimens in fact warrant permanent retention, contact the park research coordinator for further instructions)

If you propose that specimens and/or material originating from such specimens be retained permanently, they will become part of National Park Service collections. You may request that such specimens and/or material originating from such specimens be loaned to one or more non-NPS institutions for management. If you do make this request, you accept responsibility for obtaining and submitting to NPS the signature of the official at each proposed repository using the form attached as Appendix A. Note that all specimens collected and material originating from such specimens remain Federal property.

**Certification**

**I certify that this application is accurate and complete. I understand a formal study (research) proposal for new or modified studies must be provided to NPS before this application can be considered. I authorize the National Park Service to seek peer reviews of my proposal.**

Signature of principal investigator: [signature] Date: 27 Jul, 2012

| For National Park Service use only | Date received | Assigned study number | Assigned permit number |
|---|---|---|---|

**Paperwork Reduction Act Statement:** A federal agency may not conduct or sponsor, and a person is not required to respond to a collection of information unless it displays a valid OMB control number. Public reporting for this collection of information form is estimated to average 1.38 hours per response, including the time for reviewing instructions, gathering and maintaining data, and completing and reviewing the forms. Direct comments regarding this burden estimate or any aspect of this form to Dr. John G. Dennis,

Natural Resources (3130 MIB), National Park Service, 1849 C Street, N.W., Washington, DC 20240.
**Privacy Act Notice:** Scientific research, education and collecting activities within units of the National Park System that may impact parks invoke a permitting and reporting requirement per regulations at 36 CFR 1.6 (Permits), 36 CFR 2.1 (Preservation of Natural, Cultural and Archeological Resources), and 36 CFR 2.5 (Research Specimens). The National Park Service collects information about permit applicants and permittees to administer and document research, collecting, and reporting activities within parks. The information disclosed on this form is required and may result in denial of permit applications if not provided.

84630

# SPECIAL USE PERMIT

## NATURAL AREA RESERVES SYSTEM

DEPARTMENT OF LAND AND NATURAL RESOURCES
1151 PUNCHBOWL STREET, HONOLULU, HI 96813

**EFFECTIVE:** July, 1, 2011 – July 1, 2013

**RESERVES:** Manukā, Pu`u Maka`ala, Kahauale`a (currently closed due to volcanic hazard; apply directly to Big Is NARS staff), Laupāhoehoe (need to apply via US Forest Service), Pu`u O `Umi, West Maui (Līhau, Panaewa, Honokōwai, Kahakuloa Sections), Pu`u Ali`i, Mt. Ka`ala, Pahole, Ka`ena Point, Ku`ia, Hono O Na Pali

**The Board of Land and Natural Resources or its authorized representative, with the approval of the Natural Area Reserves System Commission, hereby issues this Special Use Permit in accordance with Section 195-5, Hawaii Revised Statutes; and Title 13-209-5, Department Administrative Rules, to**


Dr. Patrick M. O'Grady, Principle Investigator
Dept. of Environmental Science, Policy & Management
137 Mulford Hall # 3114
University of California Berkeley
Berkeley, CA 94720

Ms. Jessica Craft
Dr. Kari Roesch Goodman
Mr. Gordon Bennett
Dr. Brian Ort
Dr. Richard Lapoint
Ms. Elizabeth Marrack
Dr. Neal Evenhuis, Bishop Museum

Phone: (760) 520-3144 (510) 642-0662

E-mail: jcraft86@berkeley.edu ogrady@drosophilaevolution.com neale@bishopmuseum.org


**to continue to conduct surveys for, and collection of, native arthropods as part of comparative phylogenetic study of Hawaiian arthropods funded by the National Science Foundation. The research takes two main approaches: 1) comparative phylogenetic study of independent Hawaiian insect lineages and 2) investigation of population genetics of the recently formed spoon tarsus species group of Hawaiian *Drosophila*. The Natural Area Reserves are considered to be important areas for this study; many Hawaiian arthropods are host-specific, therefore one of the aims is to expand on this with genetic studies that will help to resolve some of the taxonomic and ecological issues, producing a molecular phylogeny for *Nesophrosyne species in the Cicadellidae* (leafhoppers) and *Nesosydne* species in the Delphacidae (Planthoppers); along with Diptera (Flies) in the Limoniidae: *Dicranomyia;* Dolichopodidae: *Campsicnemus* and the Eurynogaster complex; Calliphoridae: *Dyscritomyia;* Ephydridae: *Scatellaand,* and Drosophilidae, especially the *cyrtoloma* species group, the *bristle tarsus* subgroup, spoon tarsus groups, *Scaptomyza*, and invasive members of the sophophora group, using a combination of nuclear and mitochondrial markers, as well as producing a comprehensive phylogeny for the Hawaiian Drosophilidae, relating evolutionary history to the geologic history of the islands and the ecological adaptations of the various**

species groups.

STANDARD CONDITIONS:

1. Besides conditions stipulated here, the permit holder will adhere to project specifications given in the permit application.

2. Disturbance of vegetation and wildlife will be avoided as much as possible. Do not leave trails. Activities may not impede public access.

3. Reports: (a) A field report will be submitted within 5 weeks of the project's completion. (b) Results of the project, as published or unpublished reports, also will be submitted. (c) The reports will identify the Natural Area Reserve as a project site and acknowledge the special-use permit approved by the NARS Commission.

4. Precautions will be taken to prevent introduction of plants or animals not naturally present in the area. Should an infestation develop, Permit Holder is responsible for eradication by methods to be specified by NARS--whether it occurs during or after the permit period, and even though it may be only indirectly attributable to the project activities.

5. This permit is not transferable.

6. This permit does not exempt the permit holder from complying with any other applicable rule or statute.

7. The State of Hawaii shall be released and held harmless from any and all liability for injuries or death, or damage or loss of property however occurring during any activity related to this permit. **Provision of Chapters 183, 185, and 195, Hawai'i Revised Statutes, 1985, as amended, and any other laws applicable thereto, and all rules and regulations of the Department of Land and Natural Resources shall be strictly observed. Infractions or misconducts will constitute grounds for revocation of this permit and criminal prosecution. Any person whose permit has been revoked shall not be eligible to apply for another permit until the expiration of two years from the date of revocation.**

8. The Permit Holder **is responsible for notifying appropriate DOFAW/NARS Staff, prior to conducting field to inform them of exact dates for each site visit, so that they are aware; this also allows time to make arrangements for gate keys or other logistical needs.** This is particularly important to be sure that proposed site visits do not conflict with other NARS Staff or other permitted activities, as well as to be sure that areas are not closed due to weather or other hazards such as landslides, etc.

9. The Permit Holder is responsible for explaining permit terms to participants and ensuring their compliance at all times. A copy of this permit will accompany participants in the field at all times.

10. The proposed activities to be conducted in the Natural Area Reserves fall under the Division of Forestry and Wildlife's exemption list of June 12, 2008, including but not limited to **Exemption Class 5: Basic data collection, research, experimental management, and resource evaluation activities which do not result in a serious or major disturbance to an environmental resource (5-2).** Division analysis of the proposed resource management actions concluded it will provide a positive environmental benefit and will be done in a manner to have

no negative impact on the conditions that define the area. Furthermore, the cumulative impact of these actions over the duration of the permit (1 year) will not have a significant adverse impact and will have minimal or no significant effect on the environment and are exempt from the need to prepare an environmental assessment.

**SPECIAL CONDITIONS:**

11. This Permit authorizes the following personnel to accompany the above mentioned Permit Holder as part of this study: Kari Roesch Goodman; Postdoc fellow, Brian Ort, Postdoc fellow; Rick Lapoint, Postdoc fellow; Ph.D. Student; Gordon Bennett, Ph.D. candidate; Elizabeth (Lisa) Marrack, Ph.D. Student; and Jessica Craft, Ph.D. Student; additional assistants (including other Bishop Museum staff) may be added with prior notice; **this is the responsibility of the Permit Holder.**

12. This Permit authorizes collection from representative populations and/or species of the following families and/or genera of flies: Limoniidae (*Dicranomyia*); Dolichopodidae (*Campsicnemus* and Eurynogaster complex); Calliphoridae (*Dyscritomyia*); Ephydridae (Scatellaand); and Drosophilidae, especially the *cyrtoloma* species group, the bristle tarsus subgroup, spoon tarsus groups, and invasive members of the sophophora group; collections will also be made of *Nesophrosyne* species (Cicadellidae: Leafhoppers) and *Nesosydne* species (Delphacidae: Planthoppers).

13. Flies will primarily be caught live via sweeping methods (utilizing an insect net to sweep thorough foliage) or with an aspirator. Some species will be baited for using sponges soaked with fermented mushroom bait, McPhail with protein bait, and/or Multure traps with different chemical compounds. All traps will be removed from collection site at the end of the day. Care will be taken not to damage vegetation during collection. Rare and/or fragile plants such as *Cyanea* will be noted and avoided. Special care will be taken not to collect the recently listed endangered Hawaiian *Drospohila* species of Hawaiian picture wings; this particular study is focused on non-picture wing species, so close relatives of the listed species will not be collected.

14. Insects collected will be stored in 95% alcohol and either 1) pinned post-identification and removal of sample for voucher and/or DNA collection or 2) entire insect used in DNA extraction. Any insects not used in this manner will be deposited in the Bishop Museum (principal state repository); duplicate specimens may be deposited in the host or other institutions with mutual; agreement between Bishop Museum and Permit Holder.

15. Principal means of access to areas will be via 4 x 4 vehicles and hiking on foot. Should helicopter access be deemed necessary; Permit Holder is responsible for obtaining prior permission due to the fact that these areas are all part of the Conservation District, which prohibits helicopter landings without prior authorization.

16. Special considerations: Kahauale`a NAR is closed to entry; and requires separate access permission due to continuing volcanic hazard); Laupāhoehoe is now overlain by the Hawai`i Tropical Experimental Forest under the jurisdiction of the US Forest Service; a separate application needs to be made to them..

17. Permits are issued on an annual basis; if a **renewal** is required, please submit a report detailing progress to date (not just species lists) **prior** to submitting an application form detailing future plan; renewals may be delayed or denied without prior reporting. In the case of single year permits, submit a report following the permit expiration date that details what was accomplished

during the permit period.

**WILLIAM J. AILA, JR.**, Interim Chairperson,
Board of Land and Natural Resources

**PATRICK O'GRADY**
Permit Holder

# SCIENTIFIC PERMIT

## NATIVE INVERTEBRATE RESEARCH

DEPARTMENT OF LAND AND NATURAL RESOURCES
1151 PUNCHBOWL STREET, RM. 325, HONOLULU, HI 96813
PH: (808)587-0019, FAX: (808)587-0160, EMAIL: cynthia.b.king@hawaii.gov

**EFFECTIVE: 1 JULY 2012 – 1 JULY 2013**

**Endorsement No: FHM12-292**

---

**Endorsement for a Proposal to Study/Collect Native Invertebrate Animals (Exclusive of T/E Species)**
Terms & acronyms used in this document: DLNR = Department of Land and Natural Resources. DOFAW = Division of Forestry and Wildlife. NARS = Natural Area Reserves System. T/E = Threatened/Endangered (specially-protected rare native organisms).

---

**The Board of Land and Natural Resources hereby grants permission -- under the authority of: (1) Hawaii Revised Statutes (1993) Chapter 195D Conservation of Aquatic Life, Wildlife, and Land Plants, specifically Paragraph 195D-4-f License; and (2) DLNR Chapter 124 Indigenous Wildlife, Endangered and Threatened Wildlife, and Introduced Wild Birds, specifically Paragraph 13-124-4 Scientific, Propagation, and Educational Permits -- to:**

PRINCIPAL PERMIT HOLDER:
Patrick O'Grady
Assistant Professor, ESPM
University of California, Berkeley
137 Mulford Hall #3114
Berkeley, CA 94720
Phone: (510) 642-0662 Fax: (510) 643-5098
Email: ogrady@berkeley.edu

RESEARCH ASSISTANT(S):
Kari Roesch-Goodman
Gordon Bennett
Brian Ort
Elizabeth Marrack
Jessica Craft
Richard Lapoint

... to **study/collect** the following **NATIVE INVERTEBRATE** species in the amounts and with the methods/ /materials/equipment specified below. **The purpose/objectives of the study:** The proposed research takes two main approaches: (1) comparative phylogenetic study of independent Hawaiian insect lineages, and (2) investigation of the population genetics of the recently formed spoon tarsus species group of Hawaiian *Drosophila*. **Collections will be done on the islands of: Hawai'i** (Ka'ū Forest Reserve, South Kona Forest Reserve, 'Ōla'a Forest Reserve, Upper Waiākea Forest Reserve, Hilo Forest Reserve, Hamakua Forest Reserve, Mauna Kea Forest Reserve, Kohala Forest Reserve, Honua'ula Forest Reserve, Manuka NAR, Pu'u Maka'ala NAR, Laupahoehoe NAR, Pu'u 0 'Umi NAR); **Maui** (West Maui NAR, Pu'u 'Ali'i NAR); **Oahu** (Mt. Ka'ala NAR, Pahole NAR, Ka'ena Point NAR; **Kauai** (Kuia NAR, Hono o Na Pali NAR).

---

| Common name | Scientific name | No. of species |
|---|---|---|
| Various – Refer to attached list | | Various – Refer to attached list |

**Methods/Materials/Equipment:**
Sites will be accessed by 4 wheel drive vehicles and hiking. Insects will be collected via sweeping methods (utilizing an insect net to sweep through foliage) or with an aspirator. Some species will be baited using sponges soaked with fermented mushroom bait, McPhail with protein bait, and/or Multlure traps with different chemical compounds. All traps will be removed from collection sites at the end of the day. The specimens will then be placed within a labeled vial filled with 95% alcohol. Care will be taken not to damage vegetation during collection. Rare and/or fragile plants such as *Cyanea* will be noted and avoided. Special care will be taken not to collect the recently listed endangered Hawaiian *Drosophila*.

**Endorsement No: FHM12-292**

The following are general conditions/understandings:

A. This endorsement is non-transferable or assignable.

B. Each Permit Holder is individually responsible and accountable for his or her, own actions.

C. This endorsement does not in any way make the Board of Land and Natural Resources of the State of Hawaii liable for any claims of personal injury or property damage to the Principal Permittee or Sub-Permittee or their party which may occur while engaged in activities permitted under this endorsement; further, the Principal Permit Holder and Sub-Permit Holder agree to hold the State harmless against any claims of personal injury, death or property damage resulting from the activities of the Principal Permit Holder or Sub-Permit Holders.

D. Principal Permit Holder shall provide, or make available for inspection, any raw data that may be obtained under this endorsement when requested by DOFAW.

E. The endorsement shall become valid when the Principal Permit Holder has read the endorsement in-total and acknowledges understanding and agreement to abide by the conditions by signing (affixing his/her signature) in the space provided on page 4 of the document.

F. Persons in violation of the terms and conditions of this permit and/or related or appropriate laws may be subject to criminal and or administrative penalty under Hawaii Revised Statutes §183-4, §183-5, §171-6.4 §171-31.6, Hawaii Administrative Rules §13-104-3, §13-124-8, §13-195D-9, or as otherwise provided by law. Infractions or misconducts will constitute grounds for revocation of this permit and criminal prosecution. Any person whose permit has been revoked shall not be eligible to apply for another permit until the expiration of two years from the date of revocation.

G. This endorsement may be revoked for due cause (fire danger being one, violating any of the conditions being another).

H. Specimens will be deposited in Bishop Museum and/or the University of Hawaii at Manoa Insect Museum upon completion of project; duplicates may be deposited in host institution so all material is accounted for and may be located for any future research or other needs.

I. The proposed activities to be conducted fall under DOFAW's exemption list of June 12, 2008, including but not limited to **Exemption Class 5: Basic data collection, research, experimental management, and resource evaluation activities which do not result in a serious or major disturbance to an environmental resource.** Division analysis of the proposed resource management actions concluded it will provide a positive environmental benefit and will be done in a manner to have no negative impact on the conditions that define the area. Furthermore, the cumulative impact of these and similar actions over the duration of the permit (1-year) will not have a significant adverse impact and will have minimal or no significant effect on the environment and are exempt from the need to prepare an environmental assessment.

**Endorsement No: FHM12-292**

The following are special conditions/understandings:

A. The Principal Permit Holder shall submit a report of activities for the permit period, prior to expiration of permit. The reports are due to DOFAW's main office (Attn: Entomologist) no later than one month before expiration date. The reports should indicate: When, what island, what species (best guesses are acceptable), the number of specimens collected per species, and other information required by any of the conditions of this endorsement. Copies of receipts, or other indications, for any specimen deposited at Bishop Museum and other DOFAW-authorized repositories shall be attached to the reports. The last report shall summarize/compile the accomplishments for the endorsement period. Permits are granted on a calendar year basis, even in the case of multi-year projects. If renewal is needed, submit a progress report and next year's plan in advance of expiration (at least one month) to allow time for staff evaluation and processing of renewal. **Failure to submit reports and requests in advance will result in denial of further permits in the area until resolved.**

B. This endorsement authorizes the Principal Permit Holder and Sub-Permit Holders to study/collect the organisms listed on Page 1 ONLY and only in the amounts and manner specified.

C. A copy of this endorsement and a photo identification card must be carried by the Principal Permit Holder and Sub-Permit Holders while engaged in activities permitted under this endorsement.

D. **This endorsement in no way authorizes access onto property. Permission must be acquired separately.**

E. Prior to conducting study/collecting activity on DOFAW lands, the Principal Permittee and Sub-Permittees need to have permission from the appropriate DOFAW District Offices. Write to:

Kaua`i Branch Manager, Division of Forestry & Wildlife, 3060 Eiwa Street Rm. 306, Lihue, HI 96766. (808) 274-3433; fax (808) 274-3438.

O`ahu Branch Manager, Division of Forestry & Wildlife, 2135 Makiki Heights Drive, Honolulu, HI 96822. (808) 973-9778; fax (808) 973-9781.

Maui (& Moloka`i) Branch Manager, Division of Forestry & Wildlife, 54 South High Street, Wailuku, HI 96793. (808) 984-8100; fax (808) 984-8111.

Hawai`i Branch Manager, Division of Forestry & Wildlife, Mailing address: PO Box 4849, Hilo, HI 96720. Street address: 19 E. Kawili St. (808) 974-4221; fax (808) 974-4226.

F. A Special Use Permit is needed for NARS areas. Contact the NARS Commission Executive Secretary, Division of Forestry and Wildlife, 1151 Punchbowl Street Rm. 325, Honolulu, HI 96813. (808) 587-0063; fax (808) 587-0064.

G. NOTE: For the protection of the forests and other wildland areas from pest infestations (particularly weeds and plant diseases), please clean off your shoes when moving from one island to the next.

**Endorsement No: FHM12-292**

I, **PATRICK O'GRADY,** have read the general and special conditions/understandings listed on pages 2 and 3, understand them, and hereby agree to abide by them.

**PATRICK O'GRADY**
Principal Permit Holder

**PAUL J. CONRY**, Administrator
Division of Forestry and Wildlife

# SCIENTIFIC RESEARCH AND COLLECTING PERMIT

Grants permission in accordance with the attached general and special conditions

**United States Department of the Interior**
**National Park Service**

**Hawaii Volcanoes NP**

**Study#: HAVO-00312**
**Permit#: HAVO-2012-SCI-0039**
**Start Date: Aug 07, 2012**
**Expiration Date: Sep 30, 2013**
**Coop Agreement#: n/a**
**Optional Park Code: n/a**

**Name of principal investigator:**

**Name:** Patrick O'Grady **Phone:** (510) 642-0662 **Email:** ogrady@drosophilaevolution.com

**Name of institution represented:**

University of California, Berkeley

**Co-Investigators:**

| | | |
|---|---|---|
| **Name:** brian ort | **Phone:** n/a | **Email:** ort@berkeley.edu |
| **Name:** Gordon Bennett | **Phone:** n/a | **Email:** n/a |
| **Name:** Gordon Bennett | **Phone:** n/a | **Email:** n/a |
| **Name:** jessica craft | **Phone:** 7605203144 | **Email:** jcraft86@berkeley.com |
| **Name:** kari roesch goodman | **Phone:** n/a | **Email:** n/a |
| **Name:** lisa marrack | **Phone:** n/a | **Email:** n/a |

**Project title:**

A Comparative Approach to the Evolutionary Biology of Hawaiian Insects: Population Genetic and Phylogenetic Studies

**Purpose of study:**

Since the early 1960â s Hawaiian Drosophila have been used as a model system to understand the mechanics of the evolutionary process. Their biogeography is remarkable; it is an example of rapid adaptive radiation on one of the most isolated island systems in the world. The seclusion of this lineage from mainland populations makes it a particular system of importance for further study and comparative analyses. Our research focuses on synthesizing and expanding on the accumulated research from decades of study on Hawaiian Drosophila by (1) using phylogenetic approaches to comparatively examine different lineages of endemic Hawaiian insects and (2) focusing on the genetic mechanisms of speciation at the population level.

Examining the pattern, timing, and rate of diversification in other Hawaiian insect groups (Nesophrosyne leaf hoppers and limoniid craneflies) allows us to infer common evolutionary processes. As an example, a set molecular clock can be compared to phylogenetic hypotheses of independent Hawaiian lineages to infer information about the time of colonization and rates of speciation. Biogeographic patterns can also allow us to infer how intra-island colonization and subsequent speciation events have proceeded down the island chain. A population genetics approach will also be useful in understanding how species form, particular within large, rapidly evolving lineages.

We will examine seven species of the endemic spoon tarsus clade within Hawaiian Drosophila for this study. At less than 500,000 years old, this group is recent compared to other clades of Hawaiian Drosophila. The fact that several species seemed to have formed in the past half million years, implies the speciation is either recent or ongoing in this group. By observing the evolutionary forces acting on these populations, we can gain a better understanding of the effect of gene flow, hybridization, and ancestral population size on the diversification process.

**Subject/Discipline:**

Invertebrates (Insects, Other)

**Locations authorized:**

Ola'a forest

**Transportation method to research site(s):**

We will access the park via motor vehicle. Collections will be made on foot.

**Collection of the following specimens or materials, quantities, and any limitations on collecting:**

for Scaptomyza species = 5 individuals per species

for other species (Drosophila, Nesophrosyne, Campsicnemus)= 1 individual per species

no federally listed or species of concern would be collected

**Name of repository for specimens or sample materials if applicable:**

Repository type: Will be destroyed through analysis or discarded after analysis
Objects collected:
for Scaptomyza species = 5 individuals per species

for other species (Drosophila, Nesophrosyne, Campsicnemus)= 1 individual per species

**Specific conditions or restrictions (also see attached conditions):**

1-Follow the NPS General Condition and Park specific conditions for conducting research in the Park . If applicable, a copy of USFWS endangered species permit will be provided to the park. Also the permittee agrees to keep the specific location of sensitive resources confidential. Sensitive resources include threatened species, endangered species, and rare species, archeological sites, caves, fossil sites, minerals, commercially valuable resources, and sacred ceremonial sites"

2-Only persons identified in the research permit are permitted to perform tasks associated with this permit. Names added to the permit at a later time must be given to the research coordinator before they participate in any activities.

3-contact Tracy Laqua (tracy_laqua@nps.gov) or Deb Sheppard (deb_sheppard@nps.gov) regarding disposition of your collection and dissemination of findings, (typically copies of reports/thesis are stored in park library). Note that specimen collection for personal collections are not allowed. Information given to the park may be made available to the public.

4-follow sanitation protocols to avoid bringing in nonnative weed propagules and coqui frogs into natural areas and, if applicable, argentine ants to high elevation areas .

5-Prior to going in the field, check in at the Visitor Front Desk to get current information on area closures due to environmental hazards. Contact Chief Ranger Talmadge Magno (808-985-6030) for access into areas closed to the general public.

6- Prior to entering earth cracks, conducting work on high angled slopes, and/or technical climbing, all park safety conditions must be met and a safety plan approved by Chief Ranger Talmadge Magno (808-985-6030) or his designee

7-This permit does not authorize entry into any caves or lavatubes. If applicable, follow conditions for conducting work in caves (see attached). Under no circumstances should clothing, footwear or gear that was used in a White-Nose Syndrome-affected state or region be used.

8-If applicable, follow the protocols for marking and identifying plots and equipment deployed in the field . Unidentified equipment and items in the field run the risk of being removed. Make sure that unattended equipment, plots, markers (e.g. flagging) are inconspicuous from roads and trails. The PI will provide location and description information on all equipment and identifying markers (e.g. what color flagging if any, GPS location of mist nets etc) to the Research Coordinator. All such items must be removed at the end of the study period and a notice sent to the research coordinator when all items are removed from the field.

9-If applicable, obtain an administrative use backcountry permit for all overnight stays at the Backcountry Office at the Visitor Emergency Operations Center (VEOC) tel:808-985-6178

**Recommended by ~~park staff~~(name and title):**

**Reviewed by Collections Manager:**

~~Yes~~ ____ No ____

**Approved by park official:**

**Date Approved:**

8.7.12

**Title:**

Superintendent

**I Agree To All Conditions And Restrictions Of this Permit As Specified**
(Not valid unless signed and dated by the principal investigator)

(Principal investigator's signature)

8/8/2012
(Date)

**THIS PERMIT AND ATTACHED CONDITIONS AND RESTRICTIONS MUST BE CARRIED AT ALL TIMES WHILE CONDUCTING RESEARCH ACTIVITIES IN THE DESIGNATED PARK(S)**

89480

# SPECIAL USE PERMIT

## NATURAL AREA RESERVES SYSTEM

DEPARTMENT OF LAND AND NATURAL RESOURCES
1151 PUNCHBOWL STREET, HONOLULU, HI 96813

EFFECTIVE: July 1, 2013 - June 30, 2014

RESERVE(S): **Manuka, Pu`u Maka`ala, Kahuale`a (currently closed due to volcanic hazard; apply directly to Hawaii Island NARS staff), Laupahoehoe (apply with US Forest Service), Pu`u O `Umi, West Maui, Pu`u Ali`i, Ka`ala, Pahole, Ka`ena, Kui`a, Hono O Na Pali.**

**The Board of Land and Natural Resources or its authorized representative, with the approval of the Natural Area Reserves System Commission, hereby issues this Special Use Permit in accordance with Section 195-5, Hawaii Revised Statutes; and Title 13-209-5, Department Administrative Rules, to**

Dr. Patrick M. O'Grady
Dept of Environmental Science,
Policy & Management
University of California-Berkeley
137 Mulford Hall #3114
Berkeley, CA 94720

Phone: 760-520-3144/510-642-0662
Email: ogrady@drosophilaevolution.com
Email: jcraft86@berkeley.edu

Kari Roesch Goodman
Gordon Bennett
Brian Ort
Richard Lapoint
Elizabeth Marrack
Jessica Craft
Neal Evenhuis, Bishop Museum

**to continue to conduct surveys for, and collection of, native arthropods as part of comparative phylogenetic study of Hawaiian arthropods funded by the National Science Foundation. The research has 2 main approaches: 1) comparative phylogenetic study of independent Hawaiian insect lineages, and 2) investigation of population genetics of the recently formed spoon tarsus species group of Hawaiian *Drosophila*. The NARS are considered to be important areas for this study. Many Hawaiian arthropods are host-specific, therefore one of the aims is to expand this with genetic studies that will help to resolve some of the taxonomic and ecological issues, producing a molecular phylogeny for *Nesophrosyne* species in the Cicadellidae (leafhoppers), *Nesosydne* species in the Delphacidae (planthoppers), Diptera (flies) in the Limoniidae (*Dicranomyia)*, Dolichopodidae (*Campsicnemus* and the Eurynogaster complex), Calliphoridae (*Dyscritomyia)*, Ephydriadae (*Scatellaand)*, and Drosophilidae, especially the *cyrtoloma* species group, the bristle tarsus subgroup, spoon tarsus groups, *Scaptomyza,* and invasive members of the sophophora group, using a combination of nuclear and mitochondrial markers, as well as producing a comprehensive phylogeny for the Hawaiian Drosophilidae, relating evolutionary history to the geologic history of the islands and the ecological adaptations of the various species groups.**

**STANDARD CONDITIONS:**

1. Besides conditions stipulated here, the permit holder will adhere to project specifications given in the permit application.

2. Disturbance of vegetation and wildlife will be avoided as much as possible. Do not leave trails. Activities may not impede public access.

3. Reports: (a) A field report will be submitted within 5 weeks of the project's completion. (b) Results of the project, as published or unpublished reports, also will be submitted. (c) The reports will identify the Natural Area Reserve as a project site and acknowledge the special-use permit approved by the NARS Commission.

4. Precautions will be taken to prevent introduction of plants or animals not naturally present in the area. Should an infestation develop, Permit Holder is responsible for eradication by methods to be specified by NARS--whether it occurs during or after the permit period, and even though it may be only indirectly attributable to the project activities. **Permit Holder is responsible for ensuring that all clothing and gear is cleaned before entering any Natural Area Reserve.**

5. This permit is not transferable.

6. This permit does not exempt the permit holder from complying with any other applicable rule or statute.

7. The State of Hawaii shall be released and held harmless from any and all liability for injuries or death, or damage or loss of property however occurring during any activity related to this permit. **Provision of Chapters 183, 185, and 195, Hawai`i Revised Statutes, 1985, as amended, and any other laws applicable thereto, and all rules and regulations of the Department of Land and Natural Resources shall be strictly observed. Infractions or misconducts will constitute grounds for revocation of this permit and criminal prosecution. Any person whose permit has been revoked shall not be eligible to apply for another permit until the expiration of two years from the date of revocation.**

8. The Permit Holder **is responsible for notifying appropriate DOFAW/NARS Staff, prior to conducting fieldwork to inform them of exact dates for each site visit, so that they are aware; this also allows time to make arrangements for gate keys or other logistical needs.** This is particularly important to be sure that proposed site visits do not conflict with other NARS Staff or other permitted activities, as well as to be sure that areas are not closed due to weather or other hazards such as landslides, etc.

9. The Permit Holder is responsible for explaining permit terms to participants and ensuring their compliance at all times. A copy of this permit will accompany participants in the field at all times.

10. The proposed activities to be conducted in the Natural Area Reserves fall under the Division of Forestry and Wildlife's exemption list of June 12, 2008, including but not limited to **Exemption Class 5: Basic data collection, research, experimental management, and resource evaluation activities which do not result in a serious or major disturbance to**

**an environmental resource (5-2).** Division analysis of the proposed resource management actions concluded it will provide a positive environmental benefit and will be done in a manner to have no negative impact on the conditions that define the area. Furthermore, the cumulative impact of these actions over the duration of the permit (1 year) will not have a significant adverse impact and will have minimal or no significant effect on the environment and are exempt from the need to prepare an environmental assessment.

**SPECIAL CONDITIONS:**

11. This Permit authorizes the personnel listed above to collect insects at the locations listed above. Students and additional assistants (including Bishop Museum staff) may be added with prior notice once notified by the Permit Holder.

12. This Permit authorizes collection from representative populations and/or species of the following families and/or genera of flies: Limoniidae (*Dicranomyia)*, Dolichopodidae (*Campsicnemus* and Eurynogaster complex), Calliphoridae (*Dyscritomyia*); Ephydridae (Scatellaand), and Drosophilidae, especially the *cyrtoloma* species group, the bristle tarsus subgroup, spoon tarsus groups, and invasive members of the sophophora group; collection will also be made of *Nesophrosyne* species (Cicadellidae: leafhoppers) and *Nesosydne* species (Delphacidae: planthoppers).

13. Flies will primarily be caught live via sweeping methods (utilizing an insect net to sweep through foliage) or with an aspirator. Some species will be baited by using sponges soaked with fermented mushroom bait, McPhail with protein bait, and/or Multure traps with different chemical compounds. All traps will be removed from collection site at the end of the day. Care will be taken to not damage vegetation during collection. Rare and/or fragile plants such as *Cyanea* will be noted and avoided. Special care will be taken to not collect the recently listed endangered Hawaiian *Drospohila* species of Hawaiian picture wings; this particular study is focused on non-picture wing species, so close relatives of the listed species will not be collected.

14. Insects collected will be stored in 95% alcohol and either 1 )pinned post-identification and removal of sample for voucher and/or DNA collection, or 2) entire insect used in DNA extraction. Any insects not used in this manner will be deposited in the Bishop Museum (Principal State Repository); duplicate specimens may be deposited in the host or other institutions with mutual agreement between Bishop Museum and Permit Holder.

15. Principal means of access to areas will be via 4 x 4 vehicles and hiking on foot. Should helicopter access be deemed necessary, Permit Holder is responsible for obtaining prior permission due to the fact that these areas are all part of the Conservation District which prohibits helicopter landings without prior authorization.

16. Special consierations: Kahuale`a NAR is closed to entry and requires separate access permission due to continuing volcanic hazard; Laupahoehoe is now overlain by the Hawaii Experimental Topical Forest under the jurisdiction of the US Forest Service which requires a separate permit from them.

17. Permits are issued on an annual basis. If a **renewal** is required, please submit a report detailing progress to date (not just species lists) **prior** to submitting an application form detailing future plan. Renewals may be delayed or denied without prior reporting. In the case

of single year permits, submit a report following the permit expiration date that details what was accomplished during the permit period.

**WILLIAM J. AILA, JR.**, Chairperson,
Board of Land and Natural Resources

**PATRICK O'GRADY**
Permit Holder

# SCIENTIFIC PERMIT
## NATIVE INVERTEBRATE RESEARCH

DEPARTMENT OF LAND AND NATURAL RESOURCES
1151 PUNCHBOWL STREET, RM. 325, HONOLULU, HI 96813
PH: (808)587-0019, FAX: (808)587-0160, EMAIL: cynthia.b.king@hawaii.gov

**EFFECTIVE: 1 JULY 2013 – 1 JULY 2014**

**Endorsement No: FHM13-321**

---

**Endorsement for a Proposal to Study/Collect Native Invertebrate Animals (Exclusive of T/E Species)**
Terms & acronyms used in this document: DLNR = Department of Land and Natural Resources. DOFAW = Division of Forestry and Wildlife. NARS = Natural Area Reserves System. T/E = Threatened/Endangered (specially-protected rare native organisms).

---

**The Board of Land and Natural Resources hereby grants permission -- under the authority of: (1) Hawaii Revised Statutes (1993) Chapter 195D Conservation of Aquatic Life, Wildlife, and Land Plants, specifically Paragraph 195D-4-f License; and (2) DLNR Chapter 124 Indigenous Wildlife, Endangered and Threatened Wildlife, and Introduced Wild Birds, specifically Paragraph 13-124-4 Scientific, Propagation, and Educational Permits -- to:**

PRINCIPAL PERMIT HOLDER:
Patrick O'Grady
Assistant Professor, ESPM
University of California, Berkeley
137 Mulford Hall #3114
Berkeley, CA 94720
Phone: (510) 642-0662 Fax: (510) 643-5098
Email: ogrady@ berkeley.edu

RESEARCH ASSISTANT(S):
Kari Roesch-Goodman
Gordon Bennett
Brian Ort
Elizabeth Marrack
Jessica Craft
Richard Lapoint

... to **study/collect** the following **NATIVE INVERTEBRATE** species in the amounts and with the methods/ /materials/equipment specified below. **The purpose/objectives of the study:** The proposed research takes two main approaches: (1) comparative phylogenetic study of independent Hawaiian insect lineages, and (2) investigation of the population genetics of the recently formed spoon tarsus species group of Hawaiian *Drosophila*. **Collections will be done on the islands of: Hawai'i** (Ka'ū Forest Reserve, South Kona Forest Reserve, 'Ōla'a Forest Reserve, Upper Waiākea Forest Reserve, Hilo Forest Reserve, Hamakua Forest Reserve, Mauna Kea Forest Reserve, Kohala Forest Reserve, Honua'ula Forest Reserve, Manuka NAR, Pu'u Maka'ala NAR, Laupahoehoe NAR, Pu'u 0 'Umi NAR); **Maui** (West Maui NAR, Pu'u 'Ali'i NAR); **Oahu** (Mt. Ka'ala NAR, Pahole NAR, Ka'ena Point NAR; **Kauai** (Kuia NAR, Hono o Na Pali NAR).

---

| Common name | Scientific name | No. of species |
|---|---|---|
| Various – Refer to attached list | | Various – Refer to attached list |

**Methods/Materials/Equipment:**
Sites will be accessed by 4 wheel drive vehicles and hiking. Insects will be collected via sweeping methods (utilizing an insect net to sweep through foliage) or with an aspirator. Some species will be baited using sponges soaked with fermented mushroom bait, McPhail with protein bait, and/or Multlure traps with different chemical compounds. All traps will be removed from collection sites at the end of the day. The specimens will then be placed within a labeled vial filled with 95% alcohol. Care will be taken not to damage vegetation during collection. Rare and/or fragile plants such as *Cyanea* will be noted and avoided. Special care will be taken not to collect the recently listed endangered Hawaiian *Drosophila*.

**Endorsement No: FHM13-321**

The following are general conditions/understandings:

A. This endorsement is non-transferable or assignable.

B. Each Permit Holder is individually responsible and accountable for his or her, own actions.

C. This endorsement does not in any way make the Board of Land and Natural Resources of the State of Hawaii liable for any claims of personal injury or property damage to the Principal Permittee or Sub-Permittee or their party which may occur while engaged in activities permitted under this endorsement; further, the Principal Permit Holder and Sub-Permit Holder agree to hold the State harmless against any claims of personal injury, death or property damage resulting from the activities of the Principal Permit Holder or Sub-Permit Holders.

D. Principal Permit Holder shall provide, or make available for inspection, any raw data that may be obtained under this endorsement when requested by DOFAW.

E. The endorsement shall become valid when the Principal Permit Holder has read the endorsement in-total and acknowledges understanding and agreement to abide by the conditions by signing (affixing his/her signature) in the space provided on page 4 of the document.

F. Persons in violation of the terms and conditions of this permit and/or related or appropriate laws may be subject to criminal and or administrative penalty under Hawaii Revised Statutes §183-4, §183-5, §171-6.4 §171-31.6, Hawaii Administrative Rules §13-104-3, §13-124-8, §13-195D-9, or as otherwise provided by law. Infractions or misconducts will constitute grounds for revocation of this permit and criminal prosecution. Any person whose permit has been revoked shall not be eligible to apply for another permit until the expiration of two years from the date of revocation.

G. This endorsement may be revoked for due cause (fire danger being one, violating any of the conditions being another).

H. Specimens will be deposited in Bishop Museum and/or the University of Hawaii at Manoa Insect Museum upon completion of project; duplicates may be deposited in host institution so all material is accounted for and may be located for any future research or other needs.

I. The proposed activities to be conducted fall under DOFAW's exemption list of June 12, 2008, including but not limited to **Exemption Class 5: Basic data collection, research, experimental management, and resource evaluation activities which do not result in a serious or major disturbance to an environmental resource.** Division analysis of the proposed resource management actions concluded it will provide a positive environmental benefit and will be done in a manner to have no negative impact on the conditions that define the area. Furthermore, the cumulative impact of these and similar actions over the duration of the permit (1-year) will not have a significant adverse impact and will have minimal or no significant effect on the environment and are exempt from the need to prepare an environmental assessment.

**Endorsement No: FHM13-321**

The following are special conditions/understandings:

A. The Principal Permit Holder shall submit a report of activities for the permit period, prior to expiration of permit. The reports are due to DOFAW's main office (Attn: Entomologist) no later than one month before expiration date. The reports should indicate: When, what island, what species (best guesses are acceptable), the number of specimens collected per species, and other information required by any of the conditions of this endorsement. Copies of receipts, or other indications, for any specimen deposited at Bishop Museum and other DOFAW-authorized repositories shall be attached to the reports. The last report shall summarize/compile the accomplishments for the endorsement period. Permits are granted on a calendar year basis, even in the case of multi-year projects. If renewal is needed, submit a progress report and next year's plan in advance of expiration (at least one month) to allow time for staff evaluation and processing of renewal. **Failure to submit reports and requests in advance will result in denial of further permits in the area until resolved.**

B. This endorsement authorizes the Principal Permit Holder and Sub-Permit Holders to study/collect the organisms listed on Page 1 ONLY and only in the amounts and manner specified.

C. A copy of this endorsement and a photo identification card must be carried by the Principal Permit Holder and Sub-Permit Holders while engaged in activities permitted under this endorsement.

D. **This endorsement in no way authorizes access onto property. Permission must be acquired separately.**

E. Prior to conducting study/collecting activity on DOFAW lands, the Principal Permittee and Sub-Permittees need to have permission from the appropriate DOFAW District Offices. Write to:

Kaua`i Branch Manager, Division of Forestry & Wildlife, 3060 Eiwa Street Rm. 306, Lihue, HI 96766. (808) 274-3433; fax (808) 274-3438.

O`ahu Branch Manager, Division of Forestry & Wildlife, 2135 Makiki Heights Drive, Honolulu, HI 96822. (808) 973-9778; fax (808) 973-9781.

Maui (& Moloka`i) Branch Manager, Division of Forestry & Wildlife, 54 South High Street, Wailuku, HI 96793. (808) 984-8100; fax (808) 984-8111.

Hawai`i Branch Manager, Division of Forestry & Wildlife, Mailing address: PO Box 4849, Hilo, HI 96720. Street address: 19 E. Kawili St. (808) 974-4221; fax (808) 974-4226.

F. A Special Use Permit is needed for NARS areas. Contact the NARS Commission Executive Secretary, Division of Forestry and Wildlife, 1151 Punchbowl Street Rm. 325, Honolulu, HI 96813. (808) 587-0063; fax (808) 587-0064.

G. NOTE: For the protection of the forests and other wildland areas from pest infestations (particularly weeds and plant diseases), please clean off your shoes when moving from one island to the next.

**Endorsement No: FHM13-321**

I, **PATRICK O'GRADY,** have read the general and special conditions/understandings listed on pages 2 and 3, understand them, and hereby agree to abide by them.

**PATRICK O'GRADY**
Principal Permit Holder

**ROGER H. IMOTO**, Administrator
Division of Forestry and Wildlife

| *Species* | **Island** | **Quantity** (maximum per species) |
|---|---|---|
| *Drosophila adventitia* | Kauai | 5 |
| *Drosophila apoxyloma* | Molokai | 5 |
| *Drosophila arcuata* | Oahu | 5 |
| *Drosophila basimacula* | Kauai | 5 |
| *Drosophila basisetae* | Hawaii | 5 |
| *Drosophila beardsleyi* | Kauai | 5 |
| *Drosophila brevitarsus* | Oahu | 5 |
| *Drosophila bridwelli* | Oahu | 5 |
| *Drosophila canipolita* | Hawaii, Maui, Molokai, Oahu | 5 |
| *Drosophila ceratostoma* | Hawaii | 5 |
| *Drosophila clydonia* | Maui, Molokai | 5 |
| *Drosophila conformis* | Hawaii | 30 |
| *Drosophila dasycnemia* | Hawaii | 30 |
| *Drosophila diamphidiopoda* | Maui, Molokai | 5 |
| *Drosophila eximia* | Maui, Molokai | 5 |
| *Drosophila grimshawi* | Maui, Molokai | 5 |
| *Drosophila hawaiiensis* | Hawaii | 5 |
| *Drosophila hirtitarsus* | Hawaii, Maui, Molokai | 5 |
| *Drosophila hirtitibia* | Maui, Molokai, Oahu | 5 |
| *Drosophila imitator* | Oahu | 5 |
| *Drosophila incognita* | Hawaii | 30 |
| *Drosophila larifuga* | Oahu | 5 |
| *Drosophila latigena* | Hawaii, Maui, Molokai | 5 |
| *Drosophila limitata* | Maui | 5 |
| *Drosophila mitchelli* | Hawaii, Molokai | 5 |
| *Drosophila neutralis* | Hawaii | 30 |
| *Drosophila olaae* | Hawaii, Maui, Molokai | 5 |
| *Drosophila orestes* | Hawaii, Maui, Molokai, Oahu, Kauai | 5 |
| *Drosophila percnosoma* | Hawaii | 30 |
| *Drosophila praesutilis* | Oahu | 5 |
| *Drosophila propiofacies* | Hawaii | 5 |
| *Drosophila quadrisetae* | Maui, Molokai, Oahu, Kauai | 5 |
| *Drosophila seclusa* | Hawaii, Maui, Molokai | 5 |
| *Drosophila septuosa* | Hawaii | 30 |
| *Drosophila setipalpus* | Maui | 5 |
| *Drosophila sordidapex* | Hawaii | 30 |
| *Drosophila velata* | Oahu | 5 |
| *Drosophila waddingtoni* | Hawaii, Maui Nui | 70 |
| *Drosophila xuthoptera* | Maui, Molokai | 5 |

| | | |
|---|---|---|
| *Scaptomyza abberans* | Molokai | 5 |
| *Scaptomyza abrupta* | Maui | 30 |
| *Scaptomyza acronastes* | Oahu | 15 |
| *Scaptomyza adunca* | Oahu | 15 |
| *Scaptomyza affinicuspidata* | Maui | 15 |
| *Scaptomyza agrentifrons* | Kauai | 15 |
| *Scaptomyza albovittata* | Oahu | 5 |
| *Scaptomyza aloha* | Molokai | 5 |
| *Scaptomyza anechocerca* | Kauai | 15 |
| *Scaptomyza anomala* | Maui, Kauai | 30 |
| *Scaptomyza apiciguttula* | Hawaii | 15 |
| *Scaptomyza apponopusilla* | Hawaii | 15 |
| *Scaptomyza articulata* | Hawaii, Molokai | 15 |
| *Scaptomyza basiloba* | Hawaii | 15 |
| *Scaptomyza bilobata* | Kauai | 15 |
| *Scaptomyza bipars* | Kauai | 15 |
| *Scaptomyza brachycerca* | Hawaii | 15 |
| *Scaptomyza brunnimaculata* | Molokai | 5 |
| *Scaptomyza bryanti* | Hawaii, Maui | 5 |
| *Scaptomyza buccata* | Hawaii, Maui, Molokai, Oahu | 5 |
| *Scaptomyza calignosa* | Hawaii | 30 |
| *Scaptomyza camptochaites* | Maui | 15 |
| *Scaptomyza cerina* | Molokai | 5 |
| *Scaptomyza cnecosoma* | Oahu | 5 |
| *Scaptomyza concinna* | Maui | 15 |
| *Scaptomyza confusa* | Maui | 5 |
| *Scaptomyza connata* | Hawaii | 15 |
| *Scaptomyza cornuta* | Hawaii | 15 |
| *Scaptomyza crassifemur* | Maui, Molokai | 30 |
| *Scaptomyza cryptoloba* | Maui. Oahu | 15 |
| *Scaptomyza ctenophora* | Maui | 15 |
| *Scaptomyza cuspidata* | Hawaii, Maui | 30 |
| *Scaptomyza cyrtandrae* | Hawaii | 30 |
| *Scaptomyza decepta* | Kauai | 30 |
| *Scaptomyza denata* | Kauai | 15 |
| *Scaptomyza devexa* | Maui | 15 |
| *Scaptomyza diaphorocerca* | Molokai | 15 |
| *Scaptomyza domita* | Maui | 15 |
| *Scaptomyza dubautiae* | Maui | 15 |
| *Scaptomyza dubia* | Molokai, Kauai | 15 |
| *Scaptomyza eurystylata* | Hawaii, Maui, Oahu, Kauai | 15 |
| *Scaptomyza evexa* | Kauai | 15 |

| | | |
|---|---|---|
| *Scaptomyza exigua* | Hawaii, Maui | 30 |
| *Scaptomyza fastigata* | Hawaii, Maui, Molokai | 15 |
| *Scaptomyza finitima* | Oahu | 5 |
| *Scaptomyza flavida* | Molokai | 5 |
| *Scaptomyza fusciforns* | Oahu, Kauai | 5 |
| *Scaptomyza gilvivirilia* | Maui | 5 |
| *Scaptomyza hackmani* | Hawaii, Maui, Molokai | 30 |
| *Scaptomyza hamata* | Kauai | 5 |
| *Scaptomyza hardyi* | Kauai | 15 |
| *Scaptomyza inaequalis* | Hawaii, Maui, Molokai | 15 |
| *Scaptomyza inermis* | Hawaii | 15 |
| *Scaptomyza infurcula* | Hawaii | 15 |
| *Scaptomyza innotabilis* | Kauai | 15 |
| *Scaptomyza intricata* | Maui, Molokai | 30 |
| *Scaptomyza isopedon* | Hawaii, Maui | 15 |
| *Scaptomyza kauaiensis* | Kauai | 15 |
| *Scaptomyza latitergum* | Maui | 15 |
| *Scaptomyza levata* | Hawaii, Oahu, Kauai | 15 |
| *Scaptomyza lobifera* | Hawaii | 15 |
| *Scaptomyza longipecten* | Maui, Molokai | 15 |
| *Scaptomyza longipecten grisenonig* | Maui | 30 |
| *Scaptomyza longisetosa* | Hawaii, Maui, Molokai | 30 |
| *Scaptomyza mauiensis* | Hawaii, Maui, Molokai, Oahu, Kauai | 5 |
| *Scaptomyza mecocerca* | Kauai | 15 |
| *Scaptomyza mediana* | Maui | 15 |
| *Scaptomyza mediopallens* | Hawaii | 5 |
| *Scaptomyza mimula* | Molokai | 15 |
| *Scaptomyza mitchelli* | Hawaii | 5 |
| *Scaptomyza monticola* | Hawaii | 15 |
| *Scaptomyza multidenta* | Hawaii | 15 |
| *Scaptomyza mutica* | Maui | 5 |
| *Scaptomyza nasalis* | Maui, Molokai | 30 |
| *Scaptomyza neocyrtandrae* | Hawaii | 15 |
| *Scaptomyza nigrosignata* | Hawaii | 5 |
| *Scaptomyza obscuricornis* | Maui, Molokai, Oahu | 15 |
| *Scaptomyza obscurifrons* | Molokai | 15 |
| *Scaptomyza ochromata* | Maui | 15 |
| *Scaptomyza ostensa* | Maui | 15 |
| *Scaptomyza pallida* | Hawaii, Maui, Molokai, Oahu, Kauai | 30 |
| *Scaptomyza pallifrons* | Hawaii, Maui, Molokai, Kauai | 15 |
| *Scaptomyza palmae* | Hawaii, Maui, Oahu | 5 |
| *Scaptomyza paralobe* | Hawaii | 15 |

| | | |
|---|---|---|
| *Scaptomyza penicula* | Kauai | 15 |
| *Scaptomyza photophilia* | Hawaii | 15 |
| *Scaptomyza phyrysothrix* | Molokai | 15 |
| *Scaptomyza protensa* | Molokai | 15 |
| *Scaptomyza punctivena* | Maui | 15 |
| *Scaptomyza pusilla* | Hawaii, Maui, Molokai | 15 |
| *Scaptomyza quadridentata* | Oahu | 15 |
| *Scaptomyza recava* | Hawaii | 15 |
| *Scaptomyza recta* | Maui | 15 |
| *Scaptomyza reducta* | Hawaii, Maui | 30 |
| *Scaptomyza retusa* | Molokai | 15 |
| *Scaptomyza robusta* | Maui | 15 |
| *Scaptomyza rostrata* | Kaui | 15 |
| *Scaptomyza rotundiloba* | Oahu | 15 |
| *Scaptomyza scoliops* | Oahu | 30 |
| *Scaptomyza scoloplichas* | Hawaii | 15 |
| *Scaptomyza semiflava* | Maui, Molokai, Oahu | 30 |
| *Scaptomyza setiger* | Hawaii | 15 |
| *Scaptomyza setosiloba* | Hawaii | 15 |
| *Scaptomyza silcivola* | Hawaii, Maui, Molokai | 30 |
| *Scaptomyza spilota* | Maui | 15 |
| *Scaptomyza stramineifrons* | Oahu | 5 |
| *Scaptomyza striatifrons* | Kauai | 5 |
| *Scaptomyza tenuata* | Hawaii | 15 |
| *Scaptomyza trivatata* | Kauai | 15 |
| *Scaptomyza tumidula* | Hawaii | 15 |
| *Scaptomyza uliginosa* | Hawaii, Maui | 15 |
| *Scaptomyza umrbrosa* | Oahu, Kauai | 15 |
| *Scaptomyza undulata* | Hawaii | 30 |
| *Scaptomyza univitta* | Kauai | 15 |
| *Scaptomyza vagabunda* | Oahu, Kauai | 15 |
| *Scaptomyza varia* | Hawaii, Maui, Molokai | 15 |
| *Scaptomyza varifrons* | Oahu | 30 |
| *Scaptomyza varipicta* | Hawaii | 30 |
| *Scaptomyza villosa* | Kauai | 15 |
| *Scaptomyza waialeaeleae* | Kauai | 15 |

# SCIENTIFIC PERMIT

## NATIVE INVERTEBRATE RESEARCH

DEPARTMENT OF LAND AND NATURAL RESOURCES
1151 PUNCHBOWL STREET, RM. 325, HONOLULU, HI 96813
PH: (808)587-0019, FAX: (808)587-0160, EMAIL: cynthia.b.king@hawaii.gov

**EFFECTIVE: 24 OCTOBER 2014 – 24 OCTOBER 2015**

**Endorsement No: FHM15-362**

---

**Endorsement for a Proposal to Study/Collect Native Invertebrate Animals (Exclusive of T/E Species)**

Terms & acronyms used in this document: DLNR = Department of Land and Natural Resources. DOFAW = Division of Forestry and Wildlife. NARS = Natural Area Reserves System. T/E = Threatened/Endangered (specially-protected rare native organisms).

---

**The Board of Land and Natural Resources hereby grants permission -- under the authority of: (1) Hawaii Revised Statutes (1993) Chapter 195D Conservation of Aquatic Life, Wildlife, and Land Plants, specifically Paragraph 195D-4-f License; and (2) DLNR Chapter 124 Indigenous Wildlife, Endangered and Threatened Wildlife, and Introduced Wild Birds, specifically Paragraph 13-124-4 Scientific, Propagation, and Educational Permits -- to:**

PRINCIPAL PERMIT HOLDER:
Patrick O'Grady
Assistant Professor, ESPM
University of California, Berkeley
137 Mulford Hall #3114
Berkeley, CA 94720
Phone: (510) 642-0662 Fax: (510) 643-5098
Email: ogrady@ berkeley.edu

RESEARCH ASSISTANT(S):
Kari Roesch-Goodman
Gordon Bennett
Brian Ort
Elizabeth Marrack
Jessica Craft
Richard Lapoint

... to **study/collect** the following **NATIVE INVERTEBRATE** species in the amounts and with the methods/ /materials/equipment specified below. **The purpose/objectives of the study:** The proposed research takes two main approaches: (1) comparative phylogenetic study of independent Hawaiian insect lineages, and (2) investigation of the population genetics of the recently formed spoon tarsus species group of Hawaiian *Drosophila*. **Collections will be done on the islands of: Hawai'i** (Ka'ū Forest Reserve, South Kona Forest Reserve, 'Ōla'a Forest Reserve, Upper Waiākea Forest Reserve, Hilo Forest Reserve, Hamakua Forest Reserve, Mauna Kea Forest Reserve, Kohala Forest Reserve, Honua'ula Forest Reserve, Manuka NAR, Pu'u Maka'ala NAR, Laupahoehoe NAR, Pu'u 0 'Umi NAR); **Maui** (West Maui NAR, Pu'u 'Ali'i NAR); **Oahu** (Mt. Ka'ala NAR, Pahole NAR, Ka'ena Point NAR; **Kauai** (Kuia NAR, Hono o Na Pali NAR).

---

| **Common name** | **Scientific name** | **No. of species** |
|---|---|---|
| Various – Refer to attached list | | Various – Refer to attached list |

**Methods/Materials/Equipment:**
Sites will be accessed by 4 wheel drive vehicles and hiking. Insects will be collected via sweeping methods (utilizing an insect net to sweep through foliage) or with an aspirator. Some species will be baited using sponges soaked with fermented mushroom bait, McPhail with protein bait, and/or Multlure traps with different chemical compounds. All traps will be removed from collection sites at the end of the day. The specimens will then be placed within a labeled vial filled with 95% alcohol. Care will be taken not to damage vegetation during collection. Rare and/or fragile plants such as *Cyanea* will be noted and avoided. Special care will be taken not to collect the recently listed endangered Hawaiian *Drosophila*.

 **Endorsement No: FHM15-362**

The following are general conditions/understandings:

A. This endorsement is non-transferable or assignable.

B. Each Permit Holder is individually responsible and accountable for his or her, own actions.

C. This endorsement does not in any way make the Board of Land and Natural Resources of the State of Hawaii liable for any claims of personal injury or property damage to the Principal Permittee or Sub-Permittee or their party which may occur while engaged in activities permitted under this endorsement; further, the Principal Permit Holder and Sub-Permit Holder agree to hold the State harmless against any claims of personal injury, death or property damage resulting from the activities of the Principal Permit Holder or Sub-Permit Holders.

D. Principal Permit Holder shall provide, or make available for inspection, any raw data that may be obtained under this endorsement when requested by DOFAW.

E. The endorsement shall become valid when the Principal Permit Holder has read the endorsement in-total and acknowledges understanding and agreement to abide by the conditions by signing (affixing his/her signature) in the space provided on page 4 of the document.

F. Persons in violation of the terms and conditions of this permit and/or related or appropriate laws may be subject to criminal and or administrative penalty under Hawaii Revised Statutes §183-4, §183-5, §171-6.4 §171-31.6, Hawaii Administrative Rules §13-104-3, §13-124-8, §13-195D-9, or as otherwise provided by law. Infractions or misconducts will constitute grounds for revocation of this permit and criminal prosecution. Any person whose permit has been revoked shall not be eligible to apply for another permit until the expiration of two years from the date of revocation.

G. This endorsement may be revoked for due cause (fire danger being one, violating any of the conditions being another).

H. Specimens will be deposited in Bishop Museum and/or the University of Hawaii at Manoa Insect Museum upon completion of project; duplicates may be deposited in host institution so all material is accounted for and may be located for any future research or other needs.

I. The proposed activities to be conducted fall under DOFAW's exemption list of June 12, 2008, including but not limited to **Exemption Class 5: Basic data collection, research, experimental management, and resource evaluation activities which do not result in a serious or major disturbance to an environmental resource.** Division analysis of the proposed resource management actions concluded it will provide a positive environmental benefit and will be done in a manner to have no negative impact on the conditions that define the area. Furthermore, the cumulative impact of these and similar actions over the duration of the permit (1-year) will not have a significant adverse impact and will have minimal or no significant effect on the environment and are exempt from the need to prepare an environmental assessment.

The following are special conditions/understandings:

A. The Principal Permit Holder shall submit a report of activities for the permit period, prior to expiration of permit. The reports are due to DOFAW's main office (Attn: Entomologist) no later than one month before expiration date. The reports should indicate: When, what island, what species (best guesses are acceptable), the number of specimens collected per species, and other information required by any of the conditions of this endorsement. Copies of receipts, or other indications, for any specimen deposited at Bishop Museum and other DOFAW-authorized repositories shall be attached to the reports. The last report shall summarize/compile the accomplishments for the endorsement period. Permits are granted on a calendar year basis, even in the case of multi-year projects. If renewal is needed, submit a progress report and next year's plan in advance of expiration (at least one month) to allow time for staff evaluation and processing of renewal. **Failure to submit reports and requests in advance will result in denial of further permits in the area until resolved.**

B. This endorsement authorizes the Principal Permit Holder and Sub-Permit Holders to study/collect the organisms listed on Page 1 ONLY and only in the amounts and manner specified.

C. A copy of this endorsement and a photo identification card must be carried by the Principal Permit Holder and Sub-Permit Holders while engaged in activities permitted under this endorsement.

D. **This endorsement in no way authorizes access onto property. Permission must be acquired separately.**

E. Prior to conducting study/collecting activity on DOFAW lands, the Principal Permittee and Sub-Permittees need to have permission from the appropriate DOFAW District Offices. Write to:

Kaua`i Branch Manager, Division of Forestry & Wildlife, 3060 Eiwa Street Rm. 306, Lihue, HI 96766. (808) 274-3433; fax (808) 274-3438.

O`ahu Branch Manager, Division of Forestry & Wildlife, 2135 Makiki Heights Drive, Honolulu, HI 96822. (808) 973-9778; fax (808) 973-9781.

Maui (& Moloka`i) Branch Manager, Division of Forestry & Wildlife, 54 South High Street, Wailuku, HI 96793. (808) 984-8100; fax (808) 984-8111.

Hawai`i Branch Manager, Division of Forestry & Wildlife, Mailing address: PO Box 4849, Hilo, HI 96720. Street address: 19 E. Kawili St. (808) 974-4221; fax (808) 974-4226.

F. A Special Use Permit is needed for NARS areas. Contact the NARS Commission Executive Secretary, Division of Forestry and Wildlife, 1151 Punchbowl Street Rm. 325, Honolulu, HI 96813. (808) 587-0063; fax (808) 587-0064.

G. NOTE: For the protection of the forests and other wildland areas from pest infestations (particularly weeds and plant diseases), please clean off your shoes when moving from one island to the next.

**Endorsement No: <u>FHM15-362</u>**

I, **PATRICK O'GRADY,** have read the general and special conditions/understandings listed on pages 2 and 3, understand them, and hereby agree to abide by them.

---

**PATRICK O'GRADY**
Principal Permit Holder

---

**LISA J. HADWAY**, Administrator
Division of Forestry and Wildlife

# SCIENTIFIC PERMIT

## NATIVE INVERTEBRATE RESEARCH

DEPARTMENT OF LAND AND NATURAL RESOURCES
1151 PUNCHBOWL STREET, RM. 325, HONOLULU, HI 96813
PH: (808)587-0019, FAX: (808)587-0160, EMAIL: cynthia.b.king@hawaii.gov

**EFFECTIVE: 1 JANUARY 2015 – 1 JANUARY 2016**

**Endorsement No: FHM15-362**

**Endorsement for a Proposal to Study/Collect Native Invertebrate Animals (Exclusive of T/E Species)**

Terms & acronyms used in this document: DLNR = Department of Land and Natural Resources. DOFAW = Division of Forestry and Wildlife. NARS = Natural Area Reserves System. T/E = Threatened/Endangered (specially-protected rare native organisms).

**The Board of Land and Natural Resources hereby grants permission -- under the authority of: (1) Hawaii Revised Statutes (1993) Chapter 195D Conservation of Aquatic Life, Wildlife, and Land Plants, specifically Paragraph 195D-4-f License; and (2) DLNR Chapter 124 Indigenous Wildlife, Endangered and Threatened Wildlife, and Introduced Wild Birds, specifically Paragraph 13-124-4 Scientific, Propagation, and Educational Permits -- to:**

PRINCIPAL PERMIT HOLDER:
Patrick O'Grady
Assistant Professor, ESPM
University of California, Berkeley
137 Mulford Hall #3114
Berkeley, CA 94720
Phone: (510) 642-0662 Fax: (510) 643-5098
Email: ogrady@ berkeley.edu

RESEARCH ASSISTANT(S):
Kari Roesch-Goodman
Gordon Bennett
Nina Pak
Richard Lapoint

... to **study/collect** the following **NATIVE INVERTEBRATE** species in the amounts and with the methods/ /materials/equipment specified below. **The purpose/objectives of the study:** The proposed research takes two main approaches: (1) comparative phylogenetic study of independent Hawaiian insect lineages, and (2) investigation of the population genetics of the recently formed spoon tarsus species group of Hawaiian *Drosophila*. **Collections will be done on the islands of: Hawaiʻi** (Kaʻū Forest Reserve, South Kona Forest Reserve, ʻŌlaʻa Forest Reserve, Upper Waiākea Forest Reserve, Hilo Forest Reserve, Hamakua Forest Reserve, Mauna Kea Forest Reserve, Kohala Forest Reserve, Honuaʻula Forest Reserve, Manuka NAR, Pu'u Maka'ala NAR, Laupahoehoe NAR, Pu'u 0 'Umi NAR); **Maui** (West Maui NAR, Pu'u 'Ali'i NAR); **Oahu** (Mt. Ka'ala NAR, Pahole NAR, Ka'ena Point NAR; **Kauai** (Kuia NAR, Hono o Na Pali NAR).

| Common name | Scientific name | No. of species |
|---|---|---|
| Various – Refer to attached list | | Various – Refer to attached list |

**Methods/Materials/Equipment:**

Sites will be accessed by 4 wheel drive vehicles and hiking. Insects will be collected via sweeping methods (utilizing an insect net to sweep through foliage) or with an aspirator. Some species will be baited using sponges soaked with fermented mushroom bait, McPhail with protein bait, and/or Multlure traps with different chemical compounds. All traps will be removed from collection sites at the end of the day. The specimens will then be placed within a labeled vial filled with 95% alcohol. Care will be taken not to damage vegetation during collection. Rare and/or fragile plants such as *Cyanea* will be noted and avoided. Special care will be taken not to collect the recently listed endangered Hawaiian *Drosophila*.

The following are general conditions/understandings:

A. This endorsement is non-transferable or assignable.

B. Each Permit Holder is individually responsible and accountable for his or her, own actions.

C. This endorsement does not in any way make the Board of Land and Natural Resources of the State of Hawaii liable for any claims of personal injury or property damage to the Principal Permittee or Sub-Permittee or their party which may occur while engaged in activities permitted under this endorsement; further, the Principal Permit Holder and Sub-Permit Holder agree to hold the State harmless against any claims of personal injury, death or property damage resulting from the activities of the Principal Permit Holder or Sub-Permit Holders.

D. Principal Permit Holder shall provide, or make available for inspection, any raw data that may be obtained under this endorsement when requested by DOFAW.

E. The endorsement shall become valid when the Principal Permit Holder has read the endorsement in-total and acknowledges understanding and agreement to abide by the conditions by signing (affixing his/her signature) in the space provided on page 4 of the document.

F. Persons in violation of the terms and conditions of this permit and/or related or appropriate laws may be subject to criminal and or administrative penalty under Hawaii Revised Statutes §183-4, §183-5, §171-6.4 §171-31.6, Hawaii Administrative Rules §13-104-3, §13-124-8, §13-195D-9, or as otherwise provided by law. Infractions or misconducts will constitute grounds for revocation of this permit and criminal prosecution. Any person whose permit has been revoked shall not be eligible to apply for another permit until the expiration of two years from the date of revocation.

G. This endorsement may be revoked for due cause (fire danger being one, violating any of the conditions being another).

H. Specimens will be deposited in Bishop Museum and/or the University of Hawaii at Manoa Insect Museum upon completion of project; duplicates may be deposited in host institution so all material is accounted for and may be located for any future research or other needs.

I. The proposed activities to be conducted fall under DOFAW's exemption list of June 12, 2008, including but not limited to **Exemption Class 5: Basic data collection, research, experimental management, and resource evaluation activities which do not result in a serious or major disturbance to an environmental resource.** Division analysis of the proposed resource management actions concluded it will provide a positive environmental benefit and will be done in a manner to have no negative impact on the conditions that define the area. Furthermore, the cumulative impact of these and similar actions over the duration of the permit (1-year) will not have a significant adverse impact and will have minimal or no significant effect on the environment and are exempt from the need to prepare an environmental assessment.

**Endorsement No: FHM15-362**

The following are special conditions/understandings:

A. The Principal Permit Holder shall submit a report of activities for the permit period, prior to expiration of permit. The reports are due to DOFAW's main office (Attn: Entomologist) no later than one month before expiration date. The reports should indicate: When, what island, what species (best guesses are acceptable), the number of specimens collected per species, and other information required by any of the conditions of this endorsement. Copies of receipts, or other indications, for any specimen deposited at Bishop Museum and other DOFAW-authorized repositories shall be attached to the reports. The last report shall summarize/compile the accomplishments for the endorsement period. Permits are granted on a calendar year basis, even in the case of multi-year projects. If renewal is needed, submit a progress report and next year's plan in advance of expiration (at least one month) to allow time for staff evaluation and processing of renewal. **Failure to submit reports and requests in advance will result in denial of further permits in the area until resolved.**

B. This endorsement authorizes the Principal Permit Holder and Sub-Permit Holders to study/collect the organisms listed on Page 1 ONLY and only in the amounts and manner specified.

C. A copy of this endorsement and a photo identification card must be carried by the Principal Permit Holder and Sub-Permit Holders while engaged in activities permitted under this endorsement.

D. **This endorsement in no way authorizes access onto property. Permission must be acquired separately.**

E. Prior to conducting study/collecting activity on DOFAW lands, the Principal Permittee and Sub-Permittees need to have permission from the appropriate DOFAW District Offices. Write to:

Kaua`i Branch Manager, Division of Forestry & Wildlife, 3060 Eiwa Street Rm. 306, Lihue, HI 96766. (808) 274-3433; fax (808) 274-3438.

O`ahu Branch Manager, Division of Forestry & Wildlife, 2135 Makiki Heights Drive, Honolulu, HI 96822. (808) 973-9778; fax (808) 973-9781.

Maui (& Moloka`i) Branch Manager, Division of Forestry & Wildlife, 54 South High Street, Wailuku, HI 96793. (808) 984-8100; fax (808) 984-8111.

Hawai`i Branch Manager, Division of Forestry & Wildlife, Mailing address: PO Box 4849, Hilo, HI 96720. Street address: 19 E. Kawili St. (808) 974-4221; fax (808) 974-4226.

F. A Special Use Permit is needed for NARS areas. Contact the NARS Commission Executive Secretary, Division of Forestry and Wildlife, 1151 Punchbowl Street Rm. 325, Honolulu, HI 96813. (808) 587-0063; fax (808) 587-0064.

G. NOTE: For the protection of the forests and other wildland areas from pest infestations (particularly weeds and plant diseases), please clean off your shoes when moving from one island to the next.

**Endorsement No: <u>FHM15-362</u>**

I, **PATRICK O'GRADY,** have read the general and special conditions/understandings listed on pages 2 and 3, understand them, and hereby agree to abide by them.

---

**PATRICK O'GRADY**
Principal Permit Holder

---

**LISA J. HADWAY**, Administrator
Division of Forestry and Wildlife
